# Who are the male partners of adolescent girls and young women in Swaziland? Analysis of survey data from community venues across 19 DREAMS districts

**DOI:** 10.1371/journal.pone.0203208

**Published:** 2018-09-14

**Authors:** Zahra Reynolds, Ann Gottert, Erin Luben, Bheki Mamba, Patrick Shabangu, Nsindiso Dlamini, Muhle Dlamini, Sanyukta Mathur, Julie Pulerwitz

**Affiliations:** 1 MEASURE Evaluation, University of North Carolina at Chapel Hill, Chapel Hill, N.C., United States of America; 2 Population Council, HIV and AIDS Program, Washington, D.C., United States of America; 3 Institute for Health Measurement, Mbabane, Swaziland; 4 National Emergency Response Council on HIV and AIDS, Mbabane, Swaziland; 5 Swaziland National AIDS Programme, Mbabane, Swaziland; The Institute of Tropical Medicine in Antwerp, BELGIUM

## Abstract

**Background:**

Adolescent girls and young women (AGYW, ages 15–24) are at high risk of HIV in Swaziland. Understanding more about their male sexual partners can inform HIV prevention efforts for both.

**Methods:**

Using the PLACE methodology across all 19 DREAMS implementation districts, 843 men ages 20–34 were surveyed between December 2016-February 2017. Surveys were conducted at 182 venues identified by community informants as places where AGYW and men meet/socialize. Descriptive and multivariate analyses examined characteristics and risk behaviors of male partners of AGYW.

**Results:**

Men’s average age was 25.7. Sixty-three percent reported female partners ages 15–19, and 70% reported partners ages 20–24 in the last year; of those, 12% and 11% respectively had five or more such partners. Among the 568 male partners of AGYW, 36% reported consistent condom use with their current/last partner. Forty-two percent reported testing for HIV in the last year; 6% were HIV-positive, and of those, 97% were currently on treatment. One-third (37%) reported being circumcised; among uncircumcised, 81% were not considering it. In multivariate analyses, men who reported three or more AGYW partners in the last year were more likely to be HIV-positive (aOR 3.2, 95% CI 1.1,8.8). Men were also less likely to disclose their HIV status to adolescent versus older partners (aOR 0.6, 95% CI 0.4,0.9) and partners more than 5 years younger than themselves (aOR 0.6, 95% CI 0.4,0.9). Results also revealed relatively high unemployment and mobility, substantial financial responsibilities, and periodic homelessness.

**Conclusions:**

Most men identified through community venues reported relationships with AGYW, and these relationships demonstrated substantial HIV risk. Challenging life circumstances suggest structural factors may underlie some risk behaviors. Engaging men in HIV prevention and targeted health services is critical, and informant-identified community venues are promising intervention sites to reach high-risk male partners of AGYW.

## Introduction

Swaziland, a small country bordered by South Africa to its north, west and south and Mozambique to its east, has had remarkably high prevalence and incidence of HIV since the epidemic first began in Africa. The latest findings from the 2016 Swaziland HIV Incidence Measurement Survey (SHIMS) 2 Study suggest a national prevalence of 27% for people ages 15 and older, but a decline in incidence between 2011 and 2016 [1, 2]. Women continue to share a larger burden of HIV infection than men, at 32.5% and 20.4% respectively [1]. This gender disparity is also pronounced among young people ages 15–24 years: National Emergency Response Council on HIV and AIDS (NERCHA) estimates from 2015 suggest an HIV prevalence of 15% for women in this age group versus 7% for men [3]. Incidence remains highest in women aged 20–24 years and men aged 30–34 years [3], and progress towards UNAIDS 90-90-90 goals lags starkly behind among men and young people [2].

Public health researchers and practitioners have long noted that adolescent girls and young women (AGYW) are disproportionately affected by HIV in sub-Saharan Africa [4], but also that most of these HIV infections occur within the context of sexual relationships with older men which are often characterized by inequitable gender norms and lack of power by young women to negotiate condom use or refuse sex [5–7]. Recently there has been a heightened emphasis on understanding the characteristics of male sexual partners of AGYW and dynamics within these relationships that put AGYW at risk [8]. Simultaneously there has been an increasing call for greater attention to men’s low achievement of the 90-90-90 HIV care cascade in comparison with women, which perpetuates alarmingly high AIDS-related morbidity and mortality among men [9, 10].

The DREAMS Partnership, a PEPFAR-supported initiative to reduce new HIV infections among adolescent girls and young women (AGYW, ages 15–24), selected Swaziland as a priority country due to high HIV incidence and prevalence as well as related vulnerabilities among AGYW [11]. To most effectively implement DREAMS programming and similar strategies, countries are eagerly seeking locally relevant, usable data on AGYW as well as their male partners—often considered a hidden and hard-to-reach group. Critical information includes the characteristics of high-risk male partners and where they can be reached, male partners’ uptake of HIV prevention and treatment services, such as timely HIV testing, voluntary medical male circumcision (VMMC) and antiretroviral therapy (for those who are HIV-positive)–as well as key structural factors and vulnerabilities that may underlie men’s risk and preventive behaviors. The purpose of this study was to understand the above factors through surveys with male partners of AGYW in districts across the country, so that outreach and prevention activities can better reach and meet the needs of these priority populations.

## Methods

The study took place in the 19 DREAMS implementation inkhundla (sub-national districts), out of 55 total inkhundla nationwide. These represent a mix of urban, peri-urban and rural areas and are located across Swaziland’s four geographic regions. The 19 inkhundla are: Dvokodvweni, Kwaluseni, Lobamba, Lobamba Lomdzala, Ludzeludze, Manzini North, Manzini South, Maseyisini, Mbabane East, Mbabane West, Mbangweni, Mkhiweni, Motjane, Mpolonjeni, Ngwempisi, Ntfonjeni, Piggs Peak, Siphofaneni, and Sithobela.

The study employed a modified Priorities for Local AIDS Control (PLACE) approach [12, 13], a time-location sampling strategy that aims to increase understanding of the local HIV epidemic by identifying where to reach those most likely to acquire and transmit HIV, and assess service gaps among this group. The standard PLACE method was modified to focus recruitment on places where AGYW and their partners meet and socialize.

**Fig 1:**
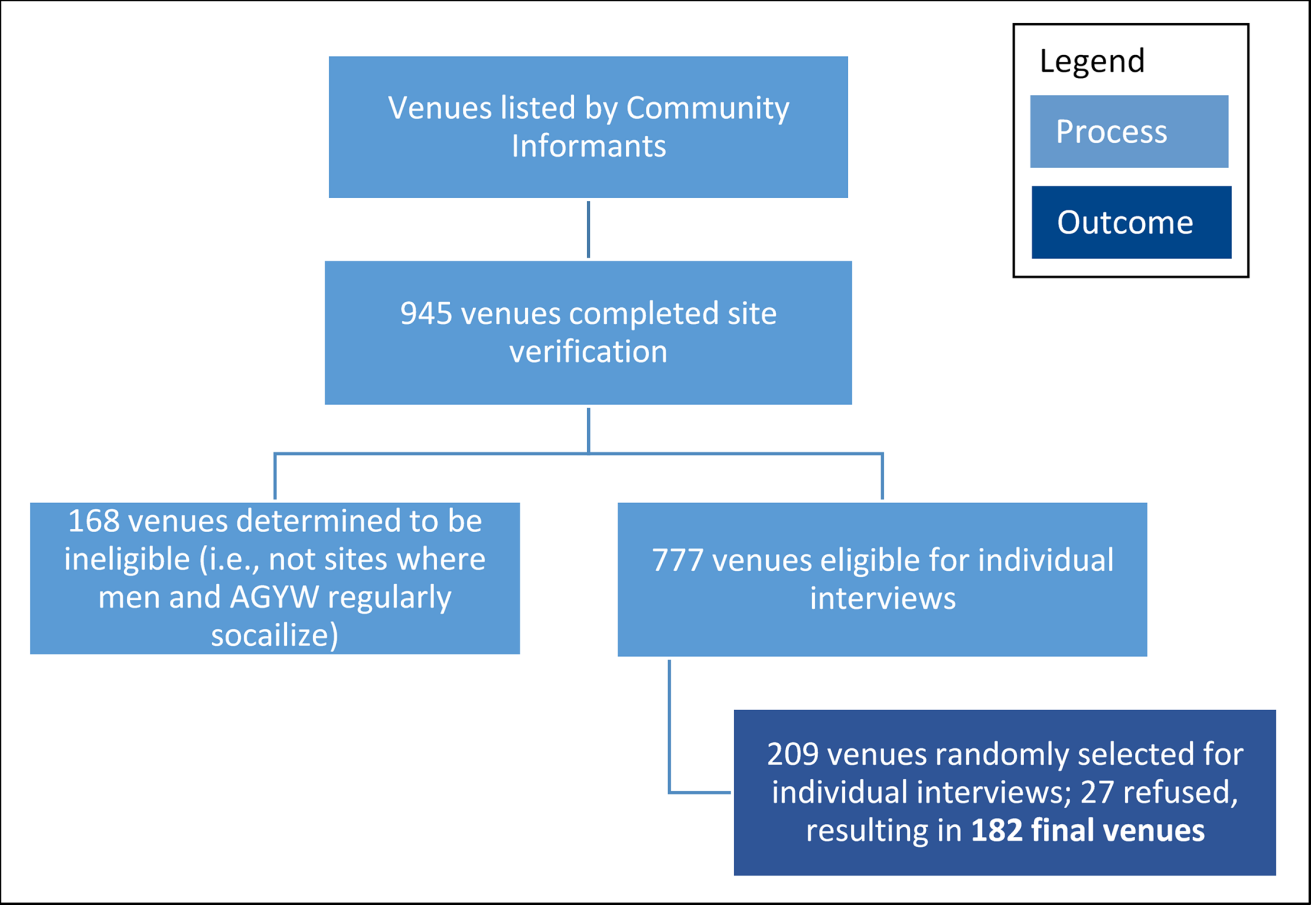
Venue selection process.

There were three main steps to data collection; a flow diagram of the process of venue selection is included in Fig 1. The first step consisted of interviews with community informants, individuals who are knowledgeable about the community in which they are found. Informants could be anyone who appeared knowledgeable such as taxi drivers, street vendors, hair dressers, community leaders, etc. In total, 1,254 community informants in the 19 inkhundla were asked where AGYW and men meet and socialize in the area. A list of 945 venues was developed based upon these responses. Each venue identified by community informants was then visited and mapped using Geographic Positioning Equipment (GPS). Of these, 168 were determined to be ineligible because informants determined they were not locations where AGYW and men regularly meet and socialize. These were most commonly informal drinking spots, kiosks/shops, and bars and clubs. More detailed information about each venue was then collected from someone knowledgeable about the venue, such as a manager or frequent patron. A final sample of venues was selected using a systematic fixed interval sampling strategy with a random start and the probability of selection proportional to the size of the venue. Ten venues were selected in each inkhundla. The size of a venue was defined by the number of people socializing at the venue during a busy time as reported by the venue representative; only venues with more than 10 patrons at their busiest time were selected for sampling. A total of 209 venues were selected in this manner, of which 37 refused to participate, resulting in a final venue sample size of 182. Types of final venues included drinking spots/‘shebeens’ (31%), kiosk/stores/shops (16%), bars/clubs (15%), parks (8%), bottle stores (4%), shisanyammas (barbeque restaurants) (3%), and churches/temples/mosques (3%).

Lastly, individual patrons and workers were interviewed at the 182 venues. In total, 843 men ages 20 to 34 were randomly sampled at the identified venues. Eligibility for men was limited to ages 20–34. This is the target age range for DREAMS-Swaziland, being the approximate age range that epidemiological evidence from the region suggests is most likely to contribute to new HIV infections and have AGYW partners [14, 15]. An additional 409 AG (ages 15–19) and 379 YW (ages 20–24) were interviewed about their own characteristics and behavior as well as those of their male partners; these data are not presented in this paper. In total, twelve individuals refused to participate, resulting in an overall refusal rate of less than one percent.

Interviews were usually conducted during a busy day and time at the venue; this was primarily weekends and evenings. Interviewers were instructed to interview 10 persons at most venues. To select potential respondents, interviewers used a random sampling strategy. At venues where there were fewer than 10 patrons, all patrons were interviewed. In the case where there were more than 10 patrons, interval sampling was employed as follows: a large “X” was conceptually drawn through the site and respondents were selected by using predetermined points in the physical space of the site along the “X”. The interval number, *i*, was calculated by counting the number along the X and dividing by the target number of interviews.

Following standard PLACE procedures, most interviews were conducted at the venue at the time of the interviewers’ visit. When necessary, the respondent was asked to move to a different location at the venue, away from their peers and others at the venue who might overhear, to preserve privacy. Interviewers were trained to only interview respondents who were not inebriated or otherwise decisionally impaired. All participation was voluntary and verbal informed consent was sought. A waiver of written consent was received from the ethical review board, as we did not want to collect the respondent’s name on any documentation.

The survey was administered in siSwati or English via tablet by experienced and trained interviewers. The survey instrument was developed based on questions commonly included in other PLACE surveys, with adaptations based on the priorities of the Swaziland PLACE study. Survey topics included demographic characteristics, employment, relationship status, fertility, residence, venue-visiting behavior, alcohol and drug use, health service utilization, sexual behavior, sexual partnerships, VMMC, and HIV testing and treatment. Data analysis was conducted using Stata 14 [16]. Results were weighted based upon the sample design, to account for sampling of venues and individuals within the venues. Results below are presented for the sample of 843 men, with a particular focus on male partners of AGYW, defined as men who reported having at least one AGYW partner in the past 12 months. Primarily descriptive analyses were carried out, since the main aim of the study was to characterize male partners’ risk and preventive behaviors. Selected multivariate analyses were also carried out, controlling for age, marital status, in-school status, and employment status, in order to more purposefully examine aspects of men’s relationships that may particularly heighten risk for AGYW.

Data were collected between December 2016 and February 2017. The study protocol was approved by the Swaziland Ethical Committee as well as the University of North Carolina Institutional Review Board.

## Results

Of the total sample of 843 men ages 20 to 34 years, nearly all (95%) were of Swazi nationality. The mean age of the respondents was 25.7 years, and 86% reported being ‘single’. Nearly two-thirds (63%) of the men reported having one or more female partners ages 15–19 in the last year; the mean age of these men was 23 years. More than two-thirds (70%) had one or more partners ages 20–24 in the last year; the mean age of these men was 27 years. Because study research questions focus primarily on characteristics of male partners of AGYW, the remaining results are presented for the sub-sample of 568 men (83% of the total sample) who reported having at least one AGYW partner (ages 15–24) in the past 12 months.

### Demographic characteristics and life contexts of male partners of AGYW

The mean age of male partners of AGYW was the same as of the total sample of men—25.7 years (Table 1). When asked about their marital status, a large proportion of male partners reported being ‘single’ (88%) versus married or cohabiting. While one in five male partners lived by themselves, 18% lived with 2–3 people, 24% with 4–5 people, and 27% lived with six or more people.

**Table 1:** Socio-demographic characteristics of male partners (MP) of AGYW.

	MP ages 20–24	MP ages 25–29	MP ages 30–34	Total
	(n = 213)	(n = 212)	(n = 143)	(n = 568)
Currently in school	32%	10%	0.4%	18%
Relationship status				
Single	98%	85%	64%	86%
Married	1%	8%	29%	9%
Cohabiting	1%	6%	5%	5%
Currently employed	32%	63%	85%	52%
Most common occupations ^a^^:^				
Small-business owner/worker	24%	25%	14%	21%
Construction worker/craftsman	27%	11%	20%	18%
Sales/shop assistant	23%	7%	7%	11%
Uniformed forces	5%	9%	16%	10%
Administrative worker	8%	11%	6%	9%
Teacher	1%	8%	12%	8%
Spent more than one month away from home in the last year	57%	50%	52%	55%
Have a regular place to stay	92%	95%	93%	93%
Slept outside due to homelessness in the past year	14%	15%	12%	15%
Jailed or imprisoned in the past year	10%	15%	13%	13%
Experienced a lack of food in the past 30 days	6%	9%	8%	8%
Hazardous/problem drinking^b^	54,8%	65.6%	70.2%	61.9%
Used marijuana in the last year	26%	30%	33%	29%
Frequency of visiting the site where interviewed:				
At least once per week	78%	75%	72%	72%
Every day	40%	37%	36%	38%
Reason for visiting the venue where interviewed ^a^:				
To socialize	78%	81%	82%	80%
To drink alcohol	34%	58%	64%	50%
To meet a new sexual partner	23%	19%	17%	20%

^a^ Respondents could select more than one response

^b^ As assessed by the AUDIT-C measure

Eighteen percent were in school at the time of the study and slightly more than half were employed (52%). Among those who were employed, 34% reported making 4000 lilangeni or more per month (approximately $304 US dollars at the time of data collection). Men who reported AGYW partners identified themselves as small-business owners/workers (21%), construction workers/craftsmen (18%), sales/shop assistants (11%), uniformed forces (10%), administrative workers (9%), or teachers (8%) (respondents could select more than one kind of occupation). Male partners of AGYW reported supporting an average of 2.4 people with their income, and a majority (57%) reported that they are the responsible party for the payment of bills at their residence. Finally, over half of respondents reported spending more than one month at a time away from home in the past year (55%); while ‘home’ was not defined within this question, respondents had just answered a series of questions about their primary place of residence.

The great majority of male partners reported having a regular place to stay (93%), but 15% said they had slept outside due to homelessness in the past year and 8% had experienced a lack of food in the past 30 days. Finally, 13% of male partners had been jailed or imprisoned in the past year.

### Site visiting behavior and alcohol and substance use

When asked specifically about the venue where the interview was conducted, 72% of male partners of AGYW reported visiting the venue at least once per week, with 38% visiting the venue every day (Table 1). When asked for their reason for visiting the venue, 80% said it was to socialize, 50% to drink alcohol, and 20% to meet a new sexual partner.

Hazardous/problem drinking, assessed via the three-item AUDIT-C measure [17], is common among male partners. Sixty-two percent fall within the definition of hazardous drinking, based on drinking frequency and quantity. Thirty-two percent of male partners said they used marijuana in the past year. Other non-prescription drug use was limited, with the next most common drug used being cocaine and opioids at 2% each (data not shown).

### Sexual relationships

Fifty-seven percent of male partners of AGYW reported having multiple (two or more) sexual partners in the last year (of any age) (Table 2). Seventy-eight percent said their partners in the past year were mostly younger in general (vs. mostly about the same age, mostly older, or both younger and older).

**Table 2:** Sexual relationships and prevention behaviors among male partners (MP) and AGYW.

	MP ages 20–24	MP ages 25–29	MP ages 30–34	Total
	(n = 213)	(n = 212)	(n = 143)	(n = 568)
Had multiple partners in past year (any age)	64%	55%	47%	**57%**
Partners were mostly younger (vs. mostly about the same age, mostly older, or both younger and older)	70%	82%	90%	**78%**
Number of AG partners in last year ^a^ (ages 15–19)				
1	44%	60%	65%	**49%**
2–4	45%	28%	29%	**39%**
5+	11%	12%	6%	**12%**
Number of YW partners in last year ^a^ (ages 20–24)				
1	51%	48%	64%	**53%**
2–4	40%	38%	27%	**36%**
5+	9%	14%	9%	**11%**
**Current/most recent sexual partner**				
Age difference				
0–4 years younger	80%	74%	47%	**71%**
5–9 years younger	3%	15%	30%	**14%**
10+ years younger	0%	1%	3%	**1%**
Uses a condom consistently	45%	31%	28%	**36%**
Used alcohol before last sex	26%	41%	35%	**33%**
Believes she has other sexual partners	47%	38%	34%	**43%**
Ever physically forced her to have sex when it was not wanted ^b^	5%	2%	5%	**4%**
Ever physically abused her ^c^	10%	13%	14%	**14%**
Talked with her about his own HIV status	47%	64%	75%	**57%**
Talked with her about her HIV status	45%	66%	74%	**57%**

^a^ Among those reporting at least one partner in this age category.

^b^ Full question was “Did you ever physically force this partner to have sex when he/she did not want to?”

^c^ Full question was “Did you ever hit, push, slap, punch, or kick this partner?”

Turning to numbers of AG and YW partners specifically, among men who reported having at least one AG partner (ages 15–19), 37% reported having one AG partner in the last year, 52% reported having 2–4 AG partners, and 11% reported having 5 or more AG partners. These rates were similar among men who had at least one YW partner in the last year: 41% had one YW partner, 48% had 2–4, and 11% had 5 or more. Younger male partners reported higher numbers of AG partners than older respondents. It is important to note that numbers of partners reported in the last year may or may not represent concurrent partnerships.

In terms of age differences with partners, over two-thirds (71%) of respondents reported that their current or most recent partner was 0 to 4 years younger than themselves, 14% 5–9 years younger, and 1% 10 or more years younger. Among the oldest group of male partners, ages 30 to 34, 30% reported that their last partner was 5 to 9 years younger and 3% 10 or more years younger.

### Risk and prevention behaviors in relationships with AGYW

When asked about their current or most recent sexual partner, one-third (36%) of male partners of AGYW reported consistent condom use with this partner; this was lowest among the highest age group (30–34), at 28% (Table 2). Four percent of respondents said they had physically forced their most recent partner to have sex when it was not wanted and 14% said they had ever physically abused their most recent partner.

Forty-three percent of male partners said they believe their current/most recent partner has other sexual partners; this was highest among young men ages 20–24. More than half of male partners had talked about their own HIV status (57%) or their partner’s HIV status (57%) with their current or most recent sexual partner.

### HIV risk perceptions and service use

When male partners of AGYW were asked how they would rate their chances of getting HIV, 48% believed they are at moderate risk, 48% at low risk, and 14% believed they have “no chance” of getting infected (Table 3).

**Table 3:** HIV risk perceptions and service use among male partners (MP) of AGYW.

	MP ages 20–24	MP ages 25–29	MP ages 30–34	Total
	(n = 213)	(n = 212)	(n = 143)	(n = 568)
Perceived risk of getting infected with HIV				
No chance	19%	10%	14%	**14%**
Low chance	50%	44%	50%	**48%**
Moderate chance	23%	34%	18%	**26%**
High chance	5%	8%	9%	**7%**
Ever tested for HIV	79%	87%	87%	**82%**
Tested for HIV in the last year	36%	46%	40%	**42%**
Circumcised	42%	34%	33%	**37%**
Considering getting circumcised (among those not, total n = 358)	12%	16%	14%	**20%**
**HIV-positive (n = 25)**	3%	6%	10%	**6%**
Currently taking antiretroviral therapy (ART)	100%	93%	100%	**97%**
Ever stopped taking ART at any point	0%	62%	9%	**30%**

Four out of five (82%) male partners reported ever having been tested for HIV. The most common reason cited for not getting tested was not wanting to know their status (41%) followed by not believing they are at risk (21%; data not shown). Forty-two percent of male partners reported testing for HIV in the last year. Most (52%) had last tested at a government clinic, health center or hospital; 13% at an NGO clinic, and 9% at a private health center (data not shown). However, among male partners ages 20–24, 23% had tested at a mobile testing unit (vs. 3% among both ages 25–29 and 30–24). About one-third (37%) of male partners reported being circumcised; this was highest in the youngest group, age 20–24, at 42%. Among uncircumcised, 80% said they were not considering it.

Six percent of male partners of AGYW reported being HIV-positive; HIV-positivity increased with age. Of the 25 HIV-positive men, 97% said they are currently taking antiretroviral therapy (ART), among whom 30% said they had stopped taking ART at some point.

### Multivariate results

In multivariate analyses that controlled for demographic characteristics (Table 4), men were less likely to talk about their own HIV status (whether negative or positive) with adolescent partners (ages 15–19 years) versus older partners (AOR 0.6, 95% CI 0.4, 0.9; p<0.01).

**Table 4:** Multivariate logistic regression results.

	aOR (95% CI)	p value	n = ^a^
**Outcome 1: Disclosed own HIV status with partner** ^b^			
Adolescent partner ages 15–19 years (vs. age 20+ years)	0.6 (0.4,0.9)	<0.01	648
Partner more than 5 years younger (vs. less than 5 years)	0.6 (0.4,0.9)	0.02	648
**Outcome 2: Perceived risk for acquiring HIV** ^c^			
Being a male partner of an AGYW	3.0 (1.8,5.1)	<0.01	729
Having 3 or more AGYW partners in the last year (vs. fewer)	1.3 (0.8, 2.1)	0.24	519
**Outcome 3: HIV-positive status**			
Having 2 or more AGYW partners in the last year (vs. fewer)	1.4 (0.6, 3.4)	0.43	407
Having 3 or more AGYW partners in the last year (vs. fewer)	3.2 (1.1, 8.8)	<0.01	411

All models controlled for respondent’s age, marital status, in-school status, and employment status.

^a^Sample sizes vary depending on survey skip patterns as well as missing values

^b^Among up to the last three partners described by the respondent (partner grid)

^c^Coded as a binary variable (moderate/high chance vs. low/no chance)

In additional multivariate analyses, being a male partner of AGYW (vs. having no recent partners age 15–24) was associated with increased perceived risk for acquiring HIV (AOR 3.0, 95% CI 1.8, 5.1; p<0.01)(moderate/high chance vs. low/no chance). Finally, men who reported three or more AGYW partners (vs. fewer) in the last year were more likely to be HIV-positive (AOR 3.2, 95% CI 1.1, 8.8; p<0.01).

## Discussion

A large proportion of 20–34 year old men, systematically selected using the well-established PLACE methodology, reported having AGYW partners, aged 15–19 (63%) and 20–24 (70%). Many of these male partners of AGYW were at substantial risk of acquiring or transmitting HIV: a majority had multiple partners in the last year as well as high total numbers of AG and YW partners, low consistency of condom use, and suboptimal levels of recent HIV testing and VMMC. Furthermore, men with several AGYW partners were more likely to be HIV-positive than men with fewer AGYW partners, and relationships with younger partners were characterized by lower discussion of HIV status than relationships with older women.

The sampling methodology used was intended to find venues with men engaged in high-risk sexual relationships, and indeed this study found a higher proportion of male partners of AGYW (as well as other men in the sample) who had multiple partners in the last year than did other studies in Swaziland. For example, according to the 2007 Demographic and Health Survey (the most recent DHS available), 29.5 percent of 20–24 year old men and 26.8 percent of 25–29 year old men had 2 or more partners in the past year (among those who had a sexual partner [18]). In comparison, the present study showed that 57% of male partners of AGYW had two or more partners in the past year. While the reasons for this discrepancy are not completely clear, it is likely that the sampling strategy was successful.

Corroborating other emerging findings from Swaziland and the southern Africa region [14, 15, 19], the present study showed that while most men tended to primarily have younger female partners, the majority have partners that are only 0–4 years younger, with a somewhat smaller proportion having partners 5–9 years younger. Only 1% reported having partners more than 10 years their junior (and only 3% among male partners ages 30–34), which may call into question popularized notions of ‘sugar daddies’ 20 or 30 years older than young women as major contributors to new HIV infections among young women [20].

Men’s reported perpetration of physical (14%) or sexual (4%) partner violence against their current or most recent partner are notable, but appear low given recent national emphasis on intimate partner violence (IPV) as a public health problem with strong links to HIV vulnerability among AGYW in Swaziland. Our findings about IPV are difficult to interpret due to a lack of recent data from Swaziland. Rates of physical violence in our study are largely consistent with a 2007 national study by UNICEF, while rates of sexual violence appear quite low [21].

Socio-demographic characteristics of the male partners of AGYW interviewed suggest structural factors that may underlie some of the HIV risk behaviors. Such factors include low marriage/cohabiting rate while still being financially responsible for supporting a large household (likely with parents, siblings, and siblings’ children), and relatively high unemployment—higher than that found in national statistics (e.g., 34% unemployment rate for men of all ages [22]). A number of other vulnerabilities–such as experiencing homelessness, having a lack of food, or being imprisoned–emerged among a substantial subsample of men. Men also experienced high mobility (likely for work or to find work), with over half having been away from home for more than one month in the last year. Recent research in the region has suggested that the types of structural factors just described strongly interact with traditional and restrictive notions of masculinity, generate substantial psychological stress among men, and can lead to men’s greater risk of acquiring and transmitting HIV [6, 23–25]. Our findings that 62% of young men are hazardous drinkers, while less surprising given that many recruitment venues were drinking establishments, nonetheless highlights alcohol abuse as a critical focus for future intervention. Finally, male partners were engaged in a range of occupations—for example in sectors such as industrial, transport and government–suggesting that HIV transmission to young women is not necessarily driven by particular occupations of their male partners, nor that certain subpopulations among these groups need to be specifically targeted for intervention.

Reported rates of HIV service use appear to be in line with findings from the Swaziland Multiple Indicator Cluster Survey 2014 showing that 55% of men ages 15–49 and 62% of men ages 15–24 were tested in the last year and know their results [19]. The reported circumcision status of 37% is higher than earlier studies,–e.g., 8% VMMC was found among men ages 15–49 years in the 2006–07 DHS [18] and 13% in a 2008 study among men ages 15–29 years—[26], suggesting substantial progress since those studies were conducted. Self-report of HIV-positive status was low at 6% compared with higher national prevalence statistics, which could be due to men in the sample being unaware of their HIV-positive status and/or to social desirability bias. The high reported prevalence of current ART use of 97% does appear in line with the most recent surveillance statistics showing that 89% of men ages 15+ nationwide are on treatment [1].

Finally, multivariate logistic regression models were used to examine ways in which AGYW may be at particular risk within their relationships. Disclosing HIV status to a partner (whether a negative, positive or unknown status) was less likely to happen when that partner was an adolescent girl, ages 15–19 years, versus older (ages 20+ years), as well as when that partner was more than five years younger than the respondent. This concurs with recent findings of attributes of age disparity in relationships that are directly responsible for placing young women at risk—including more frequent sex and less consistent condom use [5, 27], and further suggests that conversations about HIV status may be particularly difficult in relationships with adolescent girls specifically. However, few studies report on (or perhaps measure) HIV status disclosure, which the present findings suggest may be an important factor to assess and address. An additional finding from multivariate analyses was that being a male partner of AGYW (vs having no recent partners age 15–24) was associated with substantially increased perceived risk for acquiring HIV. It may be that men who have higher perceived HIV risk for HIV due to their HIV risk behaviors and partners in general, seek out AGYW partners who they may perceive to be at lower risk than older women [5, 28, 29]. However, the cross-sectional nature of the data prevents a more definitive interpretation and more research is needed to understand this finding. Finally, men who had three or more AGYW partners in the last year (vs. fewer)–a substantial subgroup of men—were more likely to be HIV-positive. Epidemiological evidence [14, 15] suggests that these men likely acquired HIV from partners closer to their same age rather than AGYW partners, and are thus currently at risk of transmitting HIV to younger partners., e.g., ages 15–24 years.

This study has several limitations worth noting. First, surveys took place in public places (although often in a more private location at those venues), which may contribute to respondents’ caution disclosing certain information particularly around stigmatized and sensitive topics such as sexual behavior and intimate partner violence. Second, the cross-sectional nature of the data does not allow for drawing causal conclusions from regression analyses presented in this paper. Finally, findings from this sample of men ages 20–34 in 19 districts may not be generalizable to men outside this age range or to other districts in Swaziland.

## Conclusion

Most of the men identified through community venues in this study reported relationships with AGYW, and high-risk male partners of AGYW visit the venue in which they were interviewed on a regular basis. Relationships were characterized by high risk for HIV acquisition and onward transmission, including high numbers of recent sexual partners, inconsistent condom use, and modest levels of preventive HIV service uptake. Specifically, relationships with girls/younger women were characterized by lower discussion of HIV status, and men with several AGYW partners were more likely to be HIV-positive. And, men’s challenging life circumstances suggest structural factors may underlie some risk behaviors. Targeted initiatives to engage high-risk men in primary prevention interventions and in diagnostic and care services that meet their needs are required to continue to reduce HIV vulnerability in Swaziland. Community venues are promising intervention sites to reach these high-risk male partners of AGYW.

## Supporting information

S1 File Data collection instruments. Forms for Community Informant Interviews, Site Verification Interviews, and Patron and Worker Interviews.
(ZIP)
