# Supplementary material for: Who are the male partners of adolescent girls and young women in Swaziland? Analysis of survey data from community venues across 19 DREAMS districts
Source: PLoS One. 2018 Sep 14;13(9):e0203208. doi: 10.1371/journal.pone.0203208 (PMC6157821; doi:10.1371/journal.pone.0203208)
Supplement: S1 File — Forms for Community Informant Interviews, Site Verification Interviews, and Patron and Worker Interviews. (ZIP) [file pone.0203208.s001.zip › Interviewer Instructions Form A 3.23.16.docx]

**Interviewer Instructions: Community Informant Interviews**

| **Steps for Conducting Community Informant Interviews** | | |  |
| --- | --- | --- | --- |
| **1** |  | Review your Tally Sheet and the types of community informants assigned to you. Make sure you know the boundaries of the zone you have been assigned for interviews. | |
| **2** |  | Upon arriving in the zone where you are working, prepare several copies of Form A by filling in your name, the date, the sequential informant number, and information about the zone where you are interviewing. Be sure to fill in one Tally Sheet for each zone you work in. | |
| **3** |  | Find a community informant to interview. Ideally, the type of informant you approach is noted on your Tally Sheet as one of your targets. If your target types of informants cannot be found, confirm with your supervisor that you can interview other types. | |
| **4** |  | Introduce yourself. Say the text on Questions to Ask Community Informants. | |
| **5** |  | Offer a Fact Sheet | |
| **6** |  | Confirm elgibility: age 18 or older and willing to answer questions | |
| **7** |  | Ask community informant to name spots, events or websites. Record this information on Form A: Spot and Event Identification Form. | |
| **8** |  | Ask community informant about each spot, event or website and record the location and how to find it, the type, busiest time and day, number of people at a busy time, whether each key population visits the spot and whether people have sex at the place. Record information on Form A. | |
| **9** |  | Thank the informant and mark the type of informant successfully interviewed on the Tally Sheet. | |

| **Questions to Ask Community Informants** Interviewers carry these instructions at all times!!    **record responses on form a: spot and event identification form** | | |
| --- | --- | --- |
| **INTRODUCE YOURSELF:**  Hello. My name is _______ and I am working with NERCHA on a study that will improve HIV prevention programs in this area. I would like to ask you some questions about where people go to meet new sexual partners around here. This should take about 10 minutes. I can offer you this Fact Sheet that has more information about the study. | | |
| **CONFIRM ELIGIBILITY:**  Are you willing to answer a few questions?  Are you at least 18 years of age? | | IF NO: STOP INTERVIEW.  IF YES: MARK THE TALLY SHEET TO INDICATE THE TYPE OF INFORMANT YOU ARE INTERVIEWING AND CONTINUE. |
| **Number on Form A** | **Question** | |
| **S1** | CONSECUTIVE NUMBER OF SPOT NAMED | |
| **S2** | **ASK INFORMANT TO NAME UP TO 10 PLACES. ASK ALL QUESTIONS HERE.**  Could you tell me where people go to meet new sex partners in this area? This includes places where people who will have sex only one time meet, but also places where people may meet partners they will know for a long time. We are interested in public places, as well as events and Internet sites. We are not interested in private homes. These places might be indoor locations where people socialize such as bars or outdoor places such as parks and streets. What are the names of these places?   - Can you tell me about any other public places where adolescent girls aged 15 to 19 socialize? - Can you tell me about public places where young women aged 20 to 24 socialize? - Can you tell me about public places where men who have sex with adolescent girls and young women socialize? - Can you tell me about public places where young men aged 20 to 34 socialize? - Can you tell me about events where men might go to meet a adolescent girls and young women? - Which websites or phone numbers do people use to meet a new sex partner? | |
| **S3** | IF INFORMANT DESCRIBES WHAT THE PLACE LOOKS LIKE OR OTHER IDENTIFYING CHARACTERISTICS OF THE PLACE, RECORD IT HERE. | |
| **S4** | In which zone/neighborhood is this place located? | |
| **S5** | What is the street address? | |
| **S6** | If you don’t know the address, can you tell me how to find the place? Are there any landmarks that would help me find the place? What is it near? | |
| **S7** | What type of place is this? ENTER CODE OF SPOT TYPE | |
| **S8** | What day of the week is the busiest at that place? | |
| **S9** | On that day, what is the busiest time? READ OPTIONS | |
| **S10** | At that time on that day, how many people visit that place? READ OPTIONS | |
| **S11** | I want to know about people who visit that place. OPTIONS: YES, NO or DON’T KNOW  Do adolescent girls aged 15 to 19 visit that place? | |
| **S12** | Do young women aged 20 to 24 visit that place? | |
| **S13** | Do men who have sex with adolescent girls and young women visit that place? | |
| **S14** | Do young men aged 20 to 34 socialize in that place? | |
| **S15** | Do people have sex at that place? | |

| SPOT TYPE CODES |  | |  | |  | |
| --- | --- | --- | --- | --- | --- | --- |
| Bar/Pub 1 | | Sports club/gym 10 | | Church/Temple/Mosque 19 | | Swimming spot 28 |
| Night club/disco 2 | | Park 11 | | School/Campus 20 | | Cultural/music event 29 |
| Massage parlor 3 | | Construction site 12 | | University 21 | | Sports event 30 |
| Brothel 4 | | Video/cinema 13 | | Tourist attraction 22 | | Funeral 31 |
| Truck stop 5 | | Kiosk/store/shop 14 | | Private house 23 | | Wedding 32 |
| Bus station/rank 6 | | Hair salon 15 | | Shisanyama 24 | | Web site 33 |
| Hotel/Guest House 7 | | Market 16 | | Bottle store 25 | | Telephone 34 |
| Sex worker street 8 | | Fast food/restaurant 17 | | Drinking spot/shebeen 26 | | Other 35 |
| Cemetery 9 | | Internet café 18 | | Abandoned buildings 27 | |  |
