# Supplementary material for: Who are the male partners of adolescent girls and young women in Swaziland? Analysis of survey data from community venues across 19 DREAMS districts
Source: PLoS One. 2018 Sep 14;13(9):e0203208. doi: 10.1371/journal.pone.0203208 (PMC6157821; doi:10.1371/journal.pone.0203208)
Supplement: S1 File — Forms for Community Informant Interviews, Site Verification Interviews, and Patron and Worker Interviews. (ZIP) [file pone.0203208.s001.zip › Swaziland FORM A 3.23.16.docx]

| **FORM A: SPOT AND EVENT IDENTIFICATION FORM** | | | | | | | | | | UP TO 10 SPOTS PER COMMUNITY INFORMANT | | | | | | | |
| --- | --- | --- | --- | --- | --- | --- | --- | --- | --- | --- | --- | --- | --- | --- | --- | --- | --- |
| A1. Interviewer Name: | | |  | | | | | A2. Interviewer Number: | | | | | |  | | | |
| A3. Date: DD/MM/YY | | | / / | | | A4. Sequential CI Number | | | | | | |  | | | | |
| A5. Region of Interview: | | | | A6. Inkundla of Interview: | | | | | | | | A7. Zone of Interview: | | | | | |
| 1. Name: | | | | 1. Name: | | | | | | | | 1. Name: | | | | | |
| 1. Code: | | | | 1. Code: | | | | | | | | 1. Code: | | | | | |
| S1. Num  **1** | S2. Spot Name | | | | | | S3. Description/Notes about the Spot | | | | | | | | | | |
| S4. Zone of Spot Location | | S5. Street Address | | | | | | | | | S6. How to find this place/landmark | | | | | | |
| **S7. Type of Spot** | **S8. Busiest Day** | **S9. Busiest Time** | | | **S10. Number at Busy Time** | | | | **Do these people visit this place?** | | | | | | **YES** | **NO** | **DK** |
| ENTER CODE FOR SPOT TYPE: | Monday 1  Tuesday 2  Wednesday 3  Thursday 4  Friday 5  Saturday 6  Sunday 7 | 11 AM to 2 PM 1  2 PM to 5 PM 2  5 PM to 8 PM 3  8 PM to 11 PM 4  11 PM to 2 AM 5  2 AM to 5 AM 6 | | | < 30 1  30 – 100 2  101 – 200 3  > 200 4 | | | | S11. Adolescent girls (15-19) | | | | | | 1 | 2 | 8 |
|  |  |  |  |  |  |  |  |  | S12. Young women (20-24) | | | | | | 1 | 2 | 8 |
|  |  |  |  |  |  |  |  |  | S13. Men who have sex with adolescent girls and young women | | | | | | 1 | 2 | 8 |
|  |  |  |  |  |  |  |  |  | S14. Men aged 20-34 years | | | | | | 1 | 2 | 8 |
|  |  |  |  |  |  |  |  |  |  | | | | | | | | |
|  |  |  |  |  |  |  |  |  | S15. Is there sex on site? | | | | | | 1 | 2 | 8 |
| S1. Num  **2** | S2. Spot Name | | | | | | S3. Description/Notes about the Spot | | | | | | | | | | |
| S4. Zone of Spot Location | | S5. Street Address | | | | | | | | | S6. How to find this place/landmark | | | | | | |
| **S7. Type of Spot** | **S8. Busiest Day** | **S9. Busiest Time** | | | **S10. Number at Busy Time** | | | | **Do these people visit this place?** | | | | | | **YES** | **NO** | **DK** |
| ENTER CODE FOR SPOT TYPE: | Monday 1  Tuesday 2  Wednesday 3  Thursday 4  Friday 5  Saturday 6  Sunday 7 | 11 AM to 2 PM 1  2 PM to 5 PM 2  5 PM to 8 PM 3  8 PM to 11 PM 4  11 PM to 2 AM 5  2 AM to 5 AM 6 | | | < 30 1  30 – 100 2  101 – 200 3  > 200 4 | | | | S11. Adolescent girls (15-19) | | | | | | 1 | 2 | 8 |
|  |  |  |  |  |  |  |  |  | S12. Young women (20-24) | | | | | | 1 | 2 | 8 |
|  |  |  |  |  |  |  |  |  | S13. Men who have sex with adolescent girls and young women | | | | | | 1 | 2 | 8 |
|  |  |  |  |  |  |  |  |  | S14. Men aged 20-34 years | | | | | | 1 | 2 | 8 |
|  |  |  |  |  |  |  |  |  |  | | | | | | | | |
|  |  |  |  |  |  |  |  |  | S15. Is there sex on site? | | | | | | 1 | 2 | 8 |

| Bar/Pub 1 | Hotel/Guest House 7 | Video/cinema 13 | Church/Temple/Mosque 19 | Bottle store 25 | Funeral 31 |
| --- | --- | --- | --- | --- | --- |
| Night club/disco 2 | Sex worker street 8 | Kiosk/store/shop 14 | School/Campus 20 | Drinking spot/shebeen 26 267 | Wedding 32 |
| Massage parlor 3 | Cemetery 9 | Hair salon 15 | University 21 | Abandoned buildings 27 | Web site 33 |
| Brothel 4 | Sports club/gym 10 | Market 16 | Tourist attraction 22 | Swimming spot 28 | Telephone 34 |
| Truck stop 5 | Park 11  Soccer Field | Fast food/restaurant 17 | Private house 23 | Cultural/music event 29 | Other 35 |
| Bus station/rank 6 | Construction site 12 | Internet café 18 | Shisanyamma 24 | Sports event 30 |  |

| S1. Num  **3** | S2. Spot Name | | | S3. Description/Notes about the Spot | | | | | |
| --- | --- | --- | --- | --- | --- | --- | --- | --- | --- |
| S4. Zone of Spot Location | | S5. Street Address | | | | S6. How to find this place/landmark | | | |
| **S7. Type of Spot** | **S8. Busiest Day** | **S9. Busiest Time** | **S10. Number at Busy Time** | | **Do these people visit this place?** | | **YES** | **NO** | **DK** |
| ENTER CODE FOR SPOT TYPE: | Monday 1  Tuesday 2  Wednesday 3  Thursday 4  Friday 5  Saturday 6  Sunday 7 | 11 AM to 2 PM 1  2 PM to 5 PM 2  5 PM to 8 PM 3  8 PM to 11 PM 4  11 PM to 2 AM 5  2 AM to 5 AM 6 | < 30 1  30 – 100 2  101 – 200 3  > 200 4 | | S11. Adolescent girls (15-19) | | 1 | 2 | 8 |
|  |  |  |  |  | S12. Young women (20-24) | | 1 | 2 | 8 |
|  |  |  |  |  | S13. Men who have sex with adolescent girls and young women | | 1 | 2 | 8 |
|  |  |  |  |  | S14. Men aged 20-34 years | | 1 | 2 | 8 |
|  |  |  |  |  |  | | | | |
|  |  |  |  |  | S15. Is there sex on site? | | 1 | 2 | 8 |
| S1. Num  **4** | S2. Spot Name | | | S3. Description/Notes about the Spot | | | | | |
| S4. Zone of Spot Location | | S5. Street Address | | | | S6. How to find this place/landmark | | | |
| **S7. Type of Spot** | **S8. Busiest Day** | **S9. Busiest Time** | **S10. Number at Busy Time** | | **Do these people visit this place?** | | **YES** | **NO** | **DK** |
| ENTER CODE FOR SPOT TYPE: | Monday 1  Tuesday 2  Wednesday 3  Thursday 4  Friday 5  Saturday 6  Sunday 7 | 11 AM to 2 PM 1  2 PM to 5 PM 2  5 PM to 8 PM 3  8 PM to 11 PM 4  11 PM to 2 AM 5  2 AM to 5 AM 6 | < 30 1  30 – 100 2  101 – 200 3  > 200 4 | | S11. Adolescent girls (15-19) | | 1 | 2 | 8 |
|  |  |  |  |  | S12. Young women (20-24) | | 1 | 2 | 8 |
|  |  |  |  |  | S13. Men who have sex with adolescent girls and young women | | 1 | 2 | 8 |
|  |  |  |  |  | S14. Men aged 20-34 years | | 1 | 2 | 8 |
|  |  |  |  |  |  | | | | |
|  |  |  |  |  | S15. Is there sex on site? | | 1 | 2 | 8 |

| Bar/Pub 1 | Hotel/Guest House 7 | Video/cinema 13 | Church/Temple/Mosque 19 | Bottle store 25 | Funeral 31 |
| --- | --- | --- | --- | --- | --- |
| Night club/disco 2 | Sex worker street 8 | Kiosk/store/shop 14 | School/Campus 20 | Drinking spot/shebeen 26 267 | Wedding 32 |
| Massage parlor 3 | Cemetery 9 | Hair salon 15 | University 21 | Abandoned buildings 27 | Web site 33 |
| Brothel 4 | Sports club/gym 10 | Market 16 | Tourist attraction 22 | Swimming spot 28 | Telephone 34 |
| Truck stop 5 | Park 11  Soccer Field | Fast food/restaurant 17 | Private house 23 | Cultural/music event 29 | Other 35 |
| Bus station/rank 6 | Construction site 12 | Internet café 18 | Shisanyamma 24 | Sports event 30 |  |
