# Supplementary material for: Who are the male partners of adolescent girls and young women in Swaziland? Analysis of survey data from community venues across 19 DREAMS districts
Source: PLoS One. 2018 Sep 14;13(9):e0203208. doi: 10.1371/journal.pone.0203208 (PMC6157821; doi:10.1371/journal.pone.0203208)
Supplement: S1 File — Forms for Community Informant Interviews, Site Verification Interviews, and Patron and Worker Interviews. (ZIP) [file pone.0203208.s001.zip › Swaziland FORM B 3 8 16.docx]

**FORM B – SPOT VERIFICATION**

| **PART 1- COMPLETE BEFORE INTERVIEW: SUPERVISOR COMPLETES SHADED AREA** | | | | | | | | | | | | | | | | | | | | | | | | | |
| --- | --- | --- | --- | --- | --- | --- | --- | --- | --- | --- | --- | --- | --- | --- | --- | --- | --- | --- | --- | --- | --- | --- | --- | --- | --- |
| B1 | SPOT ID Number | | | | | | | | | | | | | |  | | | | | | | | | | |
| B2 | Date: DD/MM/YY: | | | | | | | | | | | | | | _____/______/______ | | | | | | | | | | |
| B3 | Interviewer Code | | | | | | | | | | | | | |  | | | | | | | | | | |
| B4 | Region | | | | | | | | | | | | | |  | | | | | | | | | | |
| B5 | Inkhundla Name | | | | | | | | | | | | | |  | | | | | | | | | | |
| B6 | Inkhundla Code | | | | | | | | | | | | | |  | | | | | | | | | | |
| B7 | Zone Name | | | | | | | | | | | | | |  | | | | | | | | | | |
| B8 | Zone Code | | | | | | | | | | | | | |  | | | | | | | | | | |
| B9 | Number of community informants who named this spot | | | | | | | | | | | | | |  | | | | | | | | | | |
|  | Community Informants Reported Spot Has….. | | | | | | | | | | | | | | | | | | YES | | | | | | NO |
| B10 | Women who exchange sex for money | | | | | | | | | | | | | | | | | | 1 | | | | | | 2 |
| B11 | Adolescent girls (ages 15-19) | | | | | | | | | | | | | | | | | | 1 | | | | | | 2 |
| B12 | Young women (ages 20-24) | | | | | | | | | | | | | | | | | | 1 | | | | | | 2 |
| B13 | Men who have sex with adolescent girls and young women | | | | | | | | | | | | | | | | | | 1 | | | | | | 2 |
| Provided by community informant | | | | | | | | | Verified information by interviewer during visit | | | | | | | | | | | | | | | | |
| B14 | Spot Name: | A. | | | | | | | | | B. | | | | | | | | | | | | | | |
| B15 | Address: | A. | | | | | | | | | B. | | | | | | | | | | | | | | |
| B16 | Landmark: | A. | | | | | | | | | B. | | | | | | | | | | | | | | |
| B17 | Describe spot |  | | | | | | | | | | | | | | | | | | | | | | | |
| B18 | Type of Spot (USE CODES BELOW) | | | | | | | | | | | | | ENTER 1 CODE: | | | | | |  | | | | | |
| Bar/Pub 1 | | Sports club/gym 10 | | | | | Church/Temple/Mosque 19 | | | | | | | | | Swimming spot 28 | | | | | | | | | |
| Night club/disco 2 | | Park/field 11 | | | | | School/Campus 20 | | | | | | | | | Cultural/music event 29 | | | | | | | | | |
| Massage parlor 3 | | Construction site 12 | | | | | University 21 | | | | | | | | | Sports even 30 | | | | | | | | | |
| Brothel 4 | | Video/cinema 13 | | | | | Tourist attraction 22 | | | | | | | | | Funeral 31 | | | | | | | | | |
| Truck stop 5 | | Kiosk/store/shop 14 | | | | | Private house 23 | | | | | | | | | Wedding 32 | | | | | | | | | |
| Bus station/rank 6 | | Hair salon 15 | | | | | Shisanyamma 24 | | | | | | | | | Web site 33 | | | | | | | | | |
| Hotel/Guest House 7 | | Market 16 | | | | | Bottle store 25 | | | | | | | | | Telephone 34 | | | | | | | | | |
| Sex worker street 8 | | Fast food/restaurant 17 | | | | | Drinking spot/shebeen 26 | | | | | | | | | Other 35 | | | | | | | | | |
| Cemetery 9 | | Internet café 18 | | | | | Abandoned buildings 27 | | | | | | | | |  | | | | | | | | | |
| B19 | 1. Was the spot found?   IF SPOT WAS NOT FOUND OR NOT IN OPERATION, THEN STOP | | | | | | | Spot not found | | | | | | | | | | | | | | | 0 | | |
|  |  |  |  |  |  |  |  | Spot found and operational | | | | | | | | | | | | | | | 1 | | |
|  |  |  |  |  |  |  |  | Spot closed temporarily | | | | | | | | | | | | | | | 2 | | |
|  |  |  |  |  |  |  |  | Spot closed permanently | | | | | | | | | | | | | | | 3 | | |
|  |  |  |  |  |  |  |  | Duplicate spot (SEE 20B BELOW) | | | | | | | | | | | | | | | 4 | | |
|  |  |  |  |  |  |  |  | Other (CEE 20C BELOW) | | | | | | | | | | | | | | | 5 | | |
|  | 1. IF DUPLICATE, OTHER SPOT NUMBER | | | | | | |  | | | | | | | | | | | | | | | | | |
|  | 1. IF “OTHER” EXPLAIN | | | | | | |  | | | | | | | | | | | | | | | | | |
| B20 | GPS Coordinates in decimal degrees  USE THE GPS UNIT, WRITE COORDINATES HERE | | | | | | | | | 1. Longitude: | | | | | | | | | | | | | | | |
|  |  |  |  |  |  |  |  |  |  | 1. Latitude: | | | | | | | | | | | | | | | |
| **B21 INTERVIEWER OBSERVATION: PHYSICAL CHARACTERISTICS** | | | | | | | | | | | | | | | | | | | | | | | | | |
|  | | | | YES | NO | |  | | | | | | | | | | | | YES | | | | | | NO |
| 1. Functional electricity | | | | 1 | 2 | | 1. TV | | | | | | | | | | | | 1 | | | | | | 2 |
| 1. Tap water available | | | | 1 | 2 | | 1. Bar for alcohol sales | | | | | | | | | | | | 1 | | | | | | 2 |
| 1. Walls and ceiling | | | | 1 | 2 | | 1. Tables for visitors | | | | | | | | | | | | 1 | | | | | | 2 |
| 1. Inside toilet | | | | 1 | 2 | | 1. Beds on-site | | | | | | | | | | | | 1 | | | | | | 2 |
| 1. Spot includes outdoor area | | | | 1 | 2 | | 1. Video capability | | | | | | | | | | | | 1 | | | | | | 2 |
| **B22 INTERVIEWER OBSERVATION: HIV PREVENTION AT THE SPOT** | | | | | | | | | | | | | | | | | | | | | | | | | |
| 1. HIV/AIDS posters displayed | | | | 1 | 2 | | 1. Condom promotion posters | | | | | | | | | | | | 1 | | | | | | 2 |
| 1. Peer educators present | | | | 1 | 2 | | 1. Condoms visible | | | | | | | | | | | | 1 | | | | | | 2 |
| 1. Supportive spot manager | | | | 1 | 2 | | 1. Workplace safety notices | | | | | | | | | | | | 1 | | | | | | 2 |
| **B23 INTERVIEWER OBSERVATION OF AREA AROUND SPOT** | | | | | | | | | | | | | | | | | | | | | | | | | |
| 1. Trading center | | | | 1 | 2 | | 1. Urban slum or township | | | | | | | | | | | | 1 | | | | | | 2 |
| 1. Truck stop area | | | | 1 | 2 | | 1. Rural area | | | | | | | | | | | | 1 | | | | | | 2 |
| 1. Tourist area | | | | 1 | 2 | | 1. Plantation | | | | | | | | | | | | 1 | | | | | | 2 |
| 1. Residential area | | | | 1 | 2 | | 1. Outdoor street | | | | | | | | | | | | 1 | | | | | | 2 |
| 1. Village | | | | 1 | 2 | | 1. Commercial | | | | | | | | | | | | 1 | | | | | | 2 |
| 1. Roundabout/Big intersection | | | | 1 | 2 | | 1. Hotel complex | | | | | | | | | | | | 1 | | | | | | 1 |
| 1. Border crossing | | | | 1 | 2 | |  | | | | | | | | | | | |  | | | | | |  |
| **PART II – BEGIN INTERVIEW WITH SPOT INFORMANT** | | | | | | | | | | | | | | | | | | | | | | | | | |
| READ: Hello. My name is < > and I am working on a study coordinated by NERCHA that will improve HIV prevention programs. I would like to ask you some questions about this spot. I can offer you this information sheet that has more information about the study. This should take about 30 minutes. | | | | | | | | | | | | | | | | | | | | | | | | | |
| **NO** | **QUESTION** | | | | | | | | | | | | | | | | | | **RESPONSE** | | | | | | |
| B24 | INSTRUCTIONS TO THE INTERVIEWER (DO NOT READ ALOUD): | | | | | | | | | | | | | | | | | | YES | | | | | NO | |
|  | 1. DID YOU READ or OFFER THE FACT SHEET TO THE RESPONDENT & ANSWER QUESTIONS | | | | | | | | | | | | | | | | | | 1 | | | | | 2 | |
|  | 1. DID YOU READ THE CONSENT FORM IN LANGUAGE RESPONDENT UNDERSTANDS? | | | | | | | | | | | | | | | | | | 1 | | | | | 2 | |
| B25 | ASK: Are you willing to answer the questions I will ask you? | | | | | | | | | | | | | | | | | | 1 | | | | | 2 | |
| B26 | What is your age? IF YOUNGER THAN 18, STOP. FIND ANOTHER. | | | | | | | | | | | | | | | | AGE: | | | |  | | | | |
| IF A RESPONDENT REFUSES OR IS NOT ELIGIBLE, KEEP LOOKING FOR A KNOWLEDGEABLE RESPONDENT WHO IS ELIGIBLE AND AGREES TO PARTICIPATE. IF A RESPONDENT REFUSES BEFORE FINISHING THE INTERVIEW, FIND ANTOHER RESPONDENT TO FINISH THE INTERVIEW. | | | | | | | | | | | | | | | | | | | | | | | | | |
| B27 | Was an interview ever initiated with a willing and eligible respondent? IF NO, WHY NOT? | | | | | YES | | | | | | | | | | | | | | | 1 | | | | |
|  |  |  |  |  |  | NO (EXPLAIN): | | | | | | | | | | | | | | | 2 | | | | |
| B28 | Language of interview | | | | | | | | | | | | English | | | | | | | | 1 | | | | |
|  |  |  |  |  |  |  |  |  |  |  |  |  | SiSwati | | | | | | | | 2 | | | | |
| B29 | Sex of respondent | | | | | | | | | | | | MALE | | | | | | | | 1 | | | | |
|  |  |  |  |  |  |  |  |  |  |  |  |  | FEMALE | | | | | | | | 2 | | | | |
| B30 | Do you work here? | | | | | | | | | | | | YES | | | | | | | | 1 | | | | |
|  |  |  |  |  |  |  |  |  |  |  |  |  | NO | | | | | | | | 2 | | | | |
| B31 | How many men and women usually work here during a busy day from opening until closing? | | | | | | | | | | | 1. MEN | | | | | | | | |  | | | | |
|  |  |  |  |  |  |  |  |  |  |  |  | 1. WOMEN (15-24) | | | | | | | | |  | | | | |
| B32 | For how many years has this spot been in operation | | < 1 YEAR | | | | | | | | | | | 1 | | | | | | | | | | | |
|  |  |  | 1 – 2 YEARS | | | | | | | | | | | 2 | | | | | | | | | | | |
|  |  |  | MORE THAN 2 YEARS | | | | | | | | | | | 3 | | | | | | | | | | | |
|  |  |  | NOT APPLICABLE | | | | | | | | | | | 9 | | | | | | | | | | | |
| B33 | I have been told that people meet sexual partners at places like this. In your opinion… | |  | | | | | | | | | | | YES | | | | NO | | | | DK | | | |
|  |  |  | 1. Do women meet new male sexual partners here? | | | | | | | | | | | 1 | | | | 2 | | | | 8 | | | |
|  |  |  | 1. Do men meet new female sexual partners here? | | | | | | | | | | | 1 | | | | 2 | | | | 8 | | | |
|  |  |  | 1. Do women who have sex with men for money come here? | | | | | | | | | | | 1 | | | | 2 | | | | 8 | | | |
|  |  |  | 1. Do adolescent girls (15-19) meet sex partners here? | | | | | | | | | | | 1 | | | | 2 | | | | 8 | | | |
|  |  |  | 1. Do young women (20-24) meet sex partners here? | | | | | | | | | | | 1 | | | | 2 | | | | 8 | | | |
|  |  |  | 1. Do female staff meet new sex partners here? | | | | | | | | | | | 1 | | | | 2 | | | | 8 | | | |
|  |  |  | 1. Do people have sex at this spot? | | | | | | | | | | | 1 | | | | 2 | | | | 8 | | | |
|  |  |  | 1. Does someone here help people find sex partners? | | | | | | | | | | | 1 | | | | 2 | | | | 8 | | | |

| B34 | We want to know which days are the busiest. First, can you tell me the days that this place is closed? ENTER 9 IF CLOSED ALL DAY.  Now can you rank the remaining days/nights from busiest to least busy? What is the busiest day? CODE WITH 1. The next most busy? Continue until all days of the week are ranked. | | | | | |  | | | | | | | RANK | | | | |
| --- | --- | --- | --- | --- | --- | --- | --- | --- | --- | --- | --- | --- | --- | --- | --- | --- | --- | --- |
|  |  |  |  |  |  |  | 1. MONDAY | | | | | | |  | | | | |
|  |  |  |  |  |  |  | 1. TUESDAY | | | | | | |  | | | | |
|  |  |  |  |  |  |  | 1. WEDNESDAY | | | | | | |  | | | | |
|  |  |  |  |  |  |  | 1. THURSDAY | | | | | | |  | | | | |
|  |  |  |  |  |  |  | 1. FRIDAY | | | | | | |  | | | | |
|  |  |  |  |  |  |  | 1. SATURDAY | | | | | | |  | | | | |
|  |  |  |  |  |  |  | 1. SUNDAY | | | | | | |  | | | | |
| B35 | On the busiest day, what hours are the busiest?  CIRCLE ONLY ONE CHOICE FOR BUSIEST TIME | | | | | | 11 AM to 2 PM | | | | | | | 1 | | | | |
|  |  |  |  |  |  |  | 2 PM to 5 PM | | | | | | | 2 | | | | |
|  |  |  |  |  |  |  | 5 PM to 8 PM | | | | | | | 3 | | | | |
|  |  |  |  |  |  |  | 8 PM to 11 PM | | | | | | | 4 | | | | |
|  |  |  |  |  |  |  | 11 PM to 2 AM | | | | | | | 5 | | | | |
|  |  |  |  |  |  |  | 2 AM to 5 AM | | | | | | | 6 | | | | |
| **NO** | **QUESTION** | | | **RESPONSE** | | | | | | | | | | | | | | |
| B36 | Of young women ages 15-24 who are here at the busiest time, how many of these women in your opinion….  READ OPTIONS | | | NONE | | | | < HALF | | >= HALF | | ALMOST ALL | | | | | | DK |
|  | 1. Are under 20 years old? | | | 1 | | | | 2 | | 3 | | 4 | | | | | | 8 |
|  | 1. Drink alcohol while they are here? | | | 1 | | | | 2 | | 3 | | 4 | | | | | | 8 |
|  | 1. Are looking for a man to have sex with? | | | 1 | | | | 2 | | 3 | | 4 | | | | | | 8 |
|  | 1. Are staff who exchange sex for money with customers? | | | 1 | | | | 2 | | 3 | | 4 | | | | | | 8 |
|  | 1. Are coming here to socialize and look for a man? | | | 1 | | | | 2 | | 3 | | 4 | | | | | | 8 |
|  | 1. Go to other places around here to socialize and look for men? | | | 1 | | | | 2 | | 3 | | 4 | | | | | | 8 |
| B37 | We are also interested in knowing how often there have been HIV prevention activities at this place? For each activity, has it occurred in this spot in the past 6 months, longer than 6 months ago or never?  READ LIST |  | | | | | | | <= 6 MONTHS AGO | | MORE THAN 6 MONTHS AGO | | NEVER | | | | | DK |
|  |  | 1. Any HIV/AIDS prevention? | | | | | | | 1 | | 2 | | 3 | | | | | 8 |
|  |  | 1. Free distribution of male condoms? | | | | | | | 1 | | 2 | | 3 | | | | | 8 |
|  |  | 1. Free distribution of female condoms? | | | | | | | 1 | | 2 | | 3 | | | | | 8 |
|  |  | 1. Condoms for sale at spot? | | | | | | | 1 | | 2 | | 3 | | | | | 8 |
|  |  | 1. Persons tested onsite for HIV? | | | | | | | 1 | | 2 | | 3 | | | | | 8 |
|  |  | 1. Safer sex education by outreach workers? | | | | | | | 1 | | 2 | | 3 | | | | | 8 |
|  |  | 1. Visits by a mobile clinic? | | | | | | | 1 | | 2 | | 3 | | | | | 8 |
|  |  | | | | | ALWAYS | | | | | SOMETIMES | | | | | NEVER | | |
| B38 | In the past 6 months, how often have male condoms been available here? By available, I mean they are free or can be purchased here? | | | | | 1 | | | | | 2 | | | | | 3 | | |
| B39 | In the last 6 months, how often have female condoms been available here? By available, I mean they are free or can be purchased here? | | | | | 1 | | | | | 2 | | | | | 3 | | |
| THANK THE RESPONDENT AND END THE INTERVIEW WITH THIS RESPONDENT | | | | | | | | | | | | | | | | | | |
| **PART III – INTERVIEWER COMPLETES AFTER INTERVIEW WITH GENERAL SPOT INFORMANT** | | | | | | | | | | | | | | | | | | |
| B40 | INTERVIEWER OPINION: HOW KNOWLEDEABLE WAS THE GENERAL SPOT INFORMANT ABOUT THE ACTIVITIES AND PATRONS AT THE SPOT OF EVENT? | | | | EXTREMELY KNOWLEDGEABLE | | | | | | | | | | | | 1 | |
|  |  |  |  |  | KNOWLEDGEABLE | | | | | | | | | | | | 2 | |
|  |  |  |  |  | NOT VERY KNOWLEDGEABLE | | | | | | | | | | | | 3 | |
| B41 | TIME OF DAY | | A. HOUR: B. MINUTE | | | | | | | | | | | | C. AM/PM | | | |
