# Supplementary material for: Who are the male partners of adolescent girls and young women in Swaziland? Analysis of survey data from community venues across 19 DREAMS districts
Source: PLoS One. 2018 Sep 14;13(9):e0203208. doi: 10.1371/journal.pone.0203208 (PMC6157821; doi:10.1371/journal.pone.0203208)
Supplement: S1 File — Forms for Community Informant Interviews, Site Verification Interviews, and Patron and Worker Interviews. (ZIP) [file pone.0203208.s001.zip › Swaziland FORM C 11.6.16.docx]

**FORM C – PATRON AND WORKER INTERVIEW**

| **PART 1 – COMPLETE BEFORE INTERVIEW: SUPERVISOR COMPLETES SHADED AREA** | | | | | | | | | | | | | | |
| --- | --- | --- | --- | --- | --- | --- | --- | --- | --- | --- | --- | --- | --- | --- |
| C1 | SPOT ID Number | | | | | | | |  | | | | | |
| C2 | Date: DD/MM/YY: | | | | | | | | ____ / ____ / _____ | | | | | |
| C3 | Interviewer Code: | | | | | | | |  | | | | | |
| C4 | Region: | | | | | | | |  | | | | | |
| C5 | Inkhundla Name: | | | | | | | |  | | | | | |
| C6 | Inkhundla Code: | | | | | | | |  | | | | | |
| **PART II – BEGIN INTERVIEW WITH PATRON OR WORKER** | | | | | | | | | | | | | | |
| READ: Hello. My name is < > and I am working on a study coordinated by the < > that will improve HIV prevention programs. I would like to ask you some questions about your health. I can offer you this information sheet that has more information about the study. This should take about 30-40 minutes. | | | | | | | | | | | | | | |
| **NO** | **QUESTION** | | | | | | | | **RESPONSE** | | | | | |
|  | INSTRUCTIONS TO THE INTERVIEWER (DO NOT READ ALOUD): | | | | | | | | YES | | | | | NO |
| C7 | 1. DID YOU READ or OFFER THE FACT SHEET TO THE RESPONDENT AND ANSWER QUESTIONS? | | | | | | | | 1 | | | | | 2 |
|  | 1. DID YOU READ THE CONSENT FORM IN A LANGUAGE RESPONDENT UNDERSTANDS? | | | | | | | | 1 | | | | | 2 |
| C8 | ASK: Are you willing to answer the questions I will ask you? | | | | | | | | 1 | | | | | 2 |
| C9 | What was your sex at birth and what is your current gender? | | | | | | | | MALE | | | | | 1 |
|  |  |  |  |  |  |  |  |  | FEMALE | | | | | 2 |
|  |  |  |  |  |  |  |  |  | BORN AS MALE, NOW FEMALE | | | | | 3 |
|  |  |  |  |  |  |  |  |  | BORN AS FEMALE, NOW MALE | | | | | 4 |
| C10 | What is your age?  IF IT IS A WOMAN OUTSIDE OF THE AGES OF 15-24, STOP AND FIND ANOTHER.  IF IT IS A MAN OUTSIDE THE AGES OF 20-34, STOP AND FIND ANOTHER. | | | | | | | | AGE: | | | | |  |
| IF A RESPONDENT REFUSES OR IS NOT ELIGIBLE, KEEP LOOKING FOR AN ELIGIBLE RESPONDENT WHO AGREES TO PARTICIPATE AND BEGIN A NEW SURVEY. | | | | | | | | | | | | | | |
| C11 | Was an interview ever initiated with a willing and eligible respondent? If NO, WHY NOT | | | YES: | | | | | | | | | | 1 |
|  |  |  |  | NO (EXPLAIN): | | | | | | | | | | 2 |
| C12 | Are you currently in school? | | | | | | | | YES | | | | | 1 |
|  |  |  |  |  |  |  |  |  | NO [SKIP TO C14] | | | | | 2 |
| C13 | What is the highest level of education you have completed? | | | | | | | | Some Primary | | | | | 1 |
|  |  |  |  |  |  |  |  |  | Primary completed | | | | | 2 |
|  |  |  |  |  |  |  |  |  | Some Secondary | | | | | 3 |
|  |  |  |  |  |  |  |  |  | Secondary completed | | | | | 4 |
|  |  |  |  |  |  |  |  |  | Some high school | | | | | 5 |
|  |  |  |  |  |  |  |  |  | High school completed | | | | | 6 |
|  |  |  |  |  |  |  |  |  | Technical or vocational college completed [2 years] | | | | | 7 |
|  |  |  |  |  |  |  |  |  | University completed [3-4 years] | | | | | 8 |
|  |  |  |  |  |  |  |  |  | Postgraduate Education- Masters level or higher completed | | | | | 9 |
| C14 | Do you have a regular place to stay, where you can always sleep and where you can store your things? | | | | | | | | YES | | | | | 1 |
|  |  |  |  |  |  |  |  |  | NO [SKIP TO C17] | | | | | 2 |
| C15 | How long have you lived in your current place of residence? | | | | | | | | < 1year | | | | | 1 |
|  |  |  |  |  |  |  |  |  | 1-5 years | | | | | 2 |
|  |  |  |  |  |  |  |  |  | > 5 years | | | | | 3 |
|  |  |  |  |  |  |  |  |  | All my life | | | | | 4 |
| C16 | How many people do you live with in your place of residence? | | | | | | | | 1 | | | | | 1 |
|  |  |  |  |  |  |  |  |  | 2-3 | | | | | 2 |
|  |  |  |  |  |  |  |  |  | 4-5 | | | | | 3 |
|  |  |  |  |  |  |  |  |  | 6 or more | | | | | 4 |
| C17 | In which inkhundla do you live? | | | | | | | | Dvokodvweni | | | | | 1 |
|  |  |  |  |  |  |  |  |  | Kwaluseni | | | | | 2 |
|  |  |  |  |  |  |  |  |  | Lobamba | | | | | 3 |
|  |  |  |  |  |  |  |  |  | Lobamba Lomdzala | | | | | 4 |
|  |  |  |  |  |  |  |  |  | Ludzeludze | | | | | 5 |
|  |  |  |  |  |  |  |  |  | Manzini North | | | | | 6 |
|  |  |  |  |  |  |  |  |  | Manzini South | | | | | 7 |
|  |  |  |  |  |  |  |  |  | Maseyisini | | | | | 8 |
|  |  |  |  |  |  |  |  |  | Mbabane East | | | | | 9 |
|  |  |  |  |  |  |  |  |  | Mbabane West | | | | | 10 |
|  |  |  |  |  |  |  |  |  | Mbangweni | | | | | 11 |
|  |  |  |  |  |  |  |  |  | Mkhiweni | | | | | 12 |
|  |  |  |  |  |  |  |  |  | Motjane | | | | | 13 |
|  |  |  |  |  |  |  |  |  | Mpolonjeni | | | | | 14 |
|  |  |  |  |  |  |  |  |  | Ngwempisi | | | | | 15 |
|  |  |  |  |  |  |  |  |  | Ntfonjeni | | | | | 16 |
|  |  |  |  |  |  |  |  |  | Pigg’s Peak | | | | | 17 |
|  |  |  |  |  |  |  |  |  | Siphofaneni | | | | | 18 |
|  |  |  |  |  |  |  |  |  | Sithobela | | | | | 19 |
|  |  |  |  |  |  |  |  |  | Other: _______________ | | | | | 20 |
| C18 | Is _______ (*the Inkhundla respondent just named*) your primary place of residence (where you slept most often in the past 12 months)? | | | | | | | | Yes | | | | | 1 |
|  |  |  |  |  |  |  |  |  | No | | | | | 2 |
| C19 | In the last 12 months, have you been away from home for more than one month at a time? | | | | | | | | Yes | | | | | 1 |
|  |  |  |  |  |  |  |  |  | No | | | | | 2 |
| C20 | Are you currently working? | | | | | | | | YES, informal sector | | | | | 1 |
|  |  |  |  |  |  |  |  |  | YES, formal sector | | | | | 2 |
|  |  |  |  |  |  |  |  |  | NO, but looking for work [SKIP TO C23] | | | | | 3 |
|  |  |  |  |  |  |  |  |  | NO, and not looking for work [SKIP TO C23] | | | | | 4 |
| C21 | What is your average monthly income for yourself? | | | | | | | | Emalangeni/Rand | | | | |  |
| C22 | What do you do for work? | | | | | | | Teacher | | | | | | 1 |
|  |  |  |  |  |  |  |  | Sales/Shop Assistant | | | | | | 2 |
|  |  |  |  |  |  |  |  | Uniformed Forces (Soldiers, Police, and Corrections Officer, Fire and Emergency) | | | | | | 3 |
|  |  |  |  |  |  |  |  | Construction/craftsman | | | | | | 4 |
|  |  |  |  |  |  |  |  | Small business owner/worker/tradesman | | | | | | 5 |
|  |  |  |  |  |  |  |  | Plantation worker | | | | | | 6 |
|  |  |  |  |  |  |  |  | Forestry | | | | | | 7 |
|  |  |  |  |  |  |  |  | Mining | | | | | | 8 |
|  |  |  |  |  |  |  |  | Taxi/bus driver | | | | | | 9 |
|  |  |  |  |  |  |  |  | Taxi/Bus Conductor/Marshalls | | | | | | 10 |
|  |  |  |  |  |  |  |  | Maid/Domestic Worker | | | | | | 11 |
|  |  |  |  |  |  |  |  | Patrol Attendant | | | | | | 12 |
|  |  |  |  |  |  |  |  | Security Guards | | | | | | 13 |
|  |  |  |  |  |  |  |  | Administrative worker (govt, nonprofit, for profit) | | | | | | 14 |
|  |  |  |  |  |  |  |  | Other: _______________ | | | | | | 15 |
| C23 | During the past 12 months, where did you get most of your money? | | | | | | | Job | | | | | | 1 |
|  |  |  |  |  |  |  |  | Family | | | | | | 2 |
|  |  |  |  |  |  |  |  | Friends | | | | | | 3 |
|  |  |  |  |  |  |  |  | Girlfriend/boyfriend, partner or spouse | | | | | | 4 |
|  |  |  |  |  |  |  |  | Grants (child support, disability, etc.) | | | | | | 5 |
|  |  |  |  |  |  |  |  | Sex work | | | | | | 6 |
|  |  |  |  |  |  |  |  | Selling drugs | | | | | | 7 |
|  |  |  |  |  |  |  |  | Begging | | | | | | 8 |
|  |  |  |  |  |  |  |  | Scholarship | | | | | | 9 |
|  |  |  |  |  |  |  |  | Other:________________ | | | | | | 10 |
|  |  |  |  |  |  |  |  | I don’t have any money | | | | | | 11 |
| C24 | [WOMEN ONLY] What does your most recent partner do for work? | | | | | | Teacher | | | | | | | 1 |
|  |  |  |  |  |  |  | Sales/Shop Assistant | | | | | | | 2 |
|  |  |  |  |  |  |  | Uniformed Services (Soldiers, Police, and Corrections Officer, Fire and Emergency ) | | | | | | | 3 |
|  |  |  |  |  |  |  | Construction | | | | | | | 4 |
|  |  |  |  |  |  |  | Small business owner/worker/tradesman | | | | | | | 5 |
|  |  |  |  |  |  |  | Plantation worker | | | | | | | 6 |
|  |  |  |  |  |  |  | Forestry | | | | | | | 7 |
|  |  |  |  |  |  |  | Mining | | | | | | | 8 |
|  |  |  |  |  |  |  | Taxi/bus driver | | | | | | | 9 |
|  |  |  |  |  |  |  | Taxi/Bus Conductor/Marshalls | | | | | | | 10 |
|  |  |  |  |  |  |  | Maid/Domestic Worker | | | | | | | 11 |
|  |  |  |  |  |  |  | Patrol Attendant | | | | | | | 12 |
|  |  |  |  |  |  |  | Security Guards | | | | | | | 13 |
|  |  |  |  |  |  |  | Administrative worker (govt, nonprofit, for profit) | | | | | | | 14 |
|  |  |  |  |  |  |  | Other: _______________ | | | | | | | 15 |
|  |  | | | | | | Student | | | | | | | 16 |
|  |  | | | | | | Does not have a job | | | | | | | 17 |
| C25 | Who is responsible for the payment of bills at your place of residence? | | | | | | | | Myself | | | | | 1 |
|  |  |  |  |  |  |  |  |  | My parents | | | | | 2 |
|  |  |  |  |  |  |  |  |  | My partner/boyfriend/girlfriend/husband/wife | | | | | 3 |
|  |  |  |  |  |  |  |  |  | Family Member | | | | | 4 |
|  |  |  |  |  |  |  |  |  | Other | | | | | 5 |
| C26 | How many people do you support with your current income not including yourself? (whether living with you or elsewhere) | | | | | | | | None | | | | | 1 |
|  |  |  |  |  |  |  |  |  | 1 | | | | | 2 |
|  |  |  |  |  |  |  |  |  | 2-3 | | | | | 3 |
|  |  |  |  |  |  |  |  |  | 4-5 | | | | | 4 |
|  |  |  |  |  |  |  |  |  | 6-9 | | | | | 5 |
|  |  |  |  |  |  |  |  |  | 10 or more | | | | | 6 |
| C27 | What is your religion? | | | | | | | | Roman Catholic | | | | | 1 |
|  |  |  |  |  |  |  |  |  | Anglican | | | | | 2 |
|  |  |  |  |  |  |  |  |  | Other Christian | | | | | 3 |
|  |  |  |  |  |  |  |  |  | Muslim | | | | | 4 |
|  |  |  |  |  |  |  |  |  | Baha’i | | | | | 5 |
|  |  |  |  |  |  |  |  |  | Traditional indigenous religion | | | | | 6 |
|  |  |  |  |  |  |  |  |  | None | | | | | 7 |
|  |  |  |  |  |  |  |  |  | Other: _________________ | | | | | 8 |
| C28 | What is your marital status? | | | | | | | | Single | | | | | 1 |
|  |  |  |  |  |  |  |  |  | Married | | | | | 2 |
|  |  |  |  |  |  |  |  |  | Live in partner | | | | | 3 |
|  |  |  |  |  |  |  |  |  | Widowed | | | | | 4 |
|  |  |  |  |  |  |  |  |  | Divorced | | | | | 5 |
|  |  |  |  |  |  |  |  |  | Separated | | | | | 6 |
|  |  |  |  |  |  |  |  |  | Other | | | | | 7 |
| C29 | [MEN] If married, do you have more than one wife?  [WOMEN] If married, does your husband have other wives? | | | | | | | | YES | | | | | 1 |
|  |  |  |  |  |  |  |  |  | NO | | | | | 2 |
| C30 | [WOMEN] Have you ever been pregnant?  [MEN] Have you ever had a pregnant partner? | | | | | | | | YES | | | | | 1 |
|  |  |  |  |  |  |  |  |  | NO [SKIP to C32] | | | | | 2 |
| C31 | [WOMEN]: How old were you when you had your first child | | | | | | | | AGE: | | | | |  |
| C32 | How many children do you currently have? | | | | | | | | Number: | | | | |  |
| C33 | How many children would you like to have in your lifetime? | | | | | | | | Number: | | | | |  |
| C34 | [WOMEN] Are you currently pregnant? | | | | | | | | YES | | | | | 1 |
|  |  |  |  |  |  |  |  |  | NO | | | | | 2 |
|  |  |  |  |  |  |  |  |  | DON’T KNOW | | | | | 8 |
| C35 | What language(s) do you speak? | | |  | | | | | | | YES | | | NO |
|  |  |  |  | 1. English | | | | | | | 1 | | | 2 |
|  |  |  |  | 1. siSwati | | | | | | | 1 | | | 2 |
|  |  |  |  | 1. Other: ________________ | | | | | | | 1 | | | 2 |
| C36 | What is your nationality? | | | | | | | | Swazi | | | | | 1 |
|  |  |  |  |  |  |  |  |  | Mozambican | | | | | 2 |
|  |  |  |  |  |  |  |  |  | South African | | | | | 3 |
|  |  |  |  |  |  |  |  |  | Other: ________________ | | | | | 4 |
| C37 | Is your birth mother alive? | | | | | | | | YES [SKIP TO C39] | | | | | 1 |
|  |  |  |  |  |  |  |  |  | NO | | | | | 2 |
|  |  |  |  |  |  |  |  |  | DON’T KNOW | | | | | 8 |
| C38 | How old were you when your birth mother died? | | | | | | | | AGE (in years) | | | | |  |
|  |  |  |  |  |  |  |  |  | DON’T KNOW | | | | | 8 |
| C39 | Is your birth father alive? | | | | | | | | YES [SKIP TO C41] | | | | | 1 |
|  |  |  |  |  |  |  |  |  | NO | | | | | 2 |
|  |  |  |  |  |  |  |  |  | DON’T KNOW [SKIP to C41] | | | | | 8 |
| C40 | How old were you when your birth father died? | | | | | | | | AGE (in years) | | | | |  |
|  |  |  |  |  |  |  |  |  | DON’T KNOW | | | | | 8 |
| **PART III - SITE VISITING BEHAVIOR** | | | | | | | | | | | | | | |
| READ: Now I’m going to ask you some questions about where you socialize and how frequently. | | | | | | | | | | | | | | |
| C41 | Do you work here? | | | | | | | | YES | | | | | 1 |
|  |  |  |  |  |  |  |  |  | NO | | | | | 2 |
| C42 | How often do you visit this place where we are right now? | | | | | | | | Live at site | | | | | 1 |
|  |  |  |  |  |  |  |  |  | Daily | | | | | 2 |
|  |  |  |  |  |  |  |  |  | 4-6 times per week | | | | | 3 |
|  |  |  |  |  |  |  |  |  | 2-3 times per week | | | | | 4 |
|  |  |  |  |  |  |  |  |  | Weekly | | | | | 5 |
|  |  |  |  |  |  |  |  |  | 2-3 times per month | | | | | 6 |
|  |  |  |  |  |  |  |  |  | Monthly | | | | | 7 |
|  |  |  |  |  |  |  |  |  | Less than once per month | | | | | 8 |
|  |  |  |  |  |  |  |  |  | First time at site | | | | | 9 |
| C43 | Why did you come to this spot today? Was it… | |  | | | | | | YES | | NO | | | DK |
|  |  |  | 1. Because you work here? | | | | | | 1 | | 2 | | | 8 |
|  |  |  | 1. To socialize? | | | | | | 1 | | 2 | | | 8 |
|  |  |  | 1. To drink alcohol? | | | | | | 1 | | 2 | | | 8 |
|  |  |  | 1. To meet new people? | | | | | | 1 | | 2 | | | 8 |
|  |  |  | 1. To meet new sexual partners? | | | | | | 1 | | 2 | | | 8 |
| C44 | How many spots have you visited in the last 7 days? | | | | | | | | Number of spots: ___________ | | | | | |
| **PART IV – HEALTH AND USE OF HEALTH SERVICES** | | | | | | | | | | | | | | |
| READ: Now I’m going to ask you some questions about your health attitudes and behaviors. | | | | | | | | | | | | | | |
| C45 | In the past 3 months, have you talked to any of the following people about HIV/AIDS?  READ OPTIONS FOR EACH: | | | | | | | | | | | Yes | | No |
|  | 1. A friend or family member? | | | | | | | | | | | 1 | | 2 |
|  | 1. Peer educator trained by a program? | | | | | | | | | | | 1 | | 2 |
|  | 1. Community health worker? | | | | | | | | | | | 1 | | 2 |
|  | 1. Nurse? | | | | | | | | | | | 1 | | 2 |
|  | 1. Other? | | | | | | | | | | | 1 | | 2 |
| C46 | Do you know where to get an HIV test? | | | | | | | | | | | YES | | 1 |
|  |  |  |  |  |  |  |  |  |  |  |  | NO | | 2 |
| C47 | Have you ever been tested for HIV? | | | | | | | | | | | YES [SKIP TO C49] | | 1 |
|  |  |  |  |  |  |  |  |  |  |  |  | NO | | 2 |
| C48 | | Why have you never been tested for HIV? | | | | I DO NOT KNOW WHERE TO GO | | | | | | | | 1 |
|  |  |  |  |  |  | TESTING SITE IS TOO FAR | | | | | | | | 2 |
|  |  |  |  |  |  | GETTING TESTED IS TOO COSTLY | | | | | | | | 3 |
|  |  |  |  |  |  | I AM WORRIED THAT SOMEONE WILL SEE ME AT THE TESTING SITE | | | | | | | | 4 |
|  |  |  |  |  |  | I AM WORRIED MY RESULTS WILL NOT BE KEPT CONFIDENTIAL | | | | | | | | 5 |
|  |  |  |  |  |  | I AM WORRIED THAT HEALTH WORKERS WILL TREAT ME BADLY | | | | | | | | 6 |
|  |  |  |  |  |  | I DO NOT WANT TO KNOW MY STATUS | | | | | | | | 7 |
|  |  |  |  |  |  | I DO NOT THINK I AM AT RISK FOR HIV | | | | | | | | 8 |
|  |  |  |  |  |  | I AM WORRIED THAT TESTING POSITIVE COULD NEGATIVELY AFFECT MY CURRENT INTIMATE RELATIONSHIP(S) OR MARRIAGE | | | | | | | | 9 |
|  |  |  |  |  |  | I AM WORRIED THAT TESTING POSITIVE COULD NEGATIVELY AFFECT MY FUTURE INTIMATE RELATIONSHIP(S) OR MARRIAGE | | | | | | | | 10 |
|  |  |  |  |  |  | I AM WORRIED THAT TESTING POSITIVE COULD NEGATIVELY AFFECT MY RELATIONSHIPS WITH FAMILY AND/OR FRIENDS | | | | | | | | 11 |
|  |  |  |  |  |  | OTHER (SPECIFY): _______________ | | | | | | | | 12 |
| C49 | The last time you were tested, where were you tested for HIV? | | | | Government clinic or health center | | | | | | | | 1 | |
|  |  |  |  |  | Private health center | | | | | | | | 2 | |
|  |  |  |  |  | NGO/NGO clinic | | | | | | | | 3 | |
|  |  |  |  |  | Government hospital | | | | | | | | 4 | |
|  |  |  |  |  | Private hospital | | | | | | | | 5 | |
|  |  |  |  |  | Private doctor | | | | | | | | 6 | |
|  |  |  |  |  | Mobile testing unit | | | | | | | | 7 | |
|  |  |  |  |  | Home-based testing | | | | | | | | 8 | |
|  |  |  |  |  | Workplace | | | | | | | | 9 | |
|  |  |  |  |  | Don’t know | | | | | | | | 99 | |
| C50 | | Have you ever received your results from an HIV test? | | | | | | | | YES | | | | 1 |
|  |  |  |  |  |  |  |  |  |  | NO [SKIP TO C52] | | | | 2 |
| C51 | | When was the last time you received results from your HIV test? If you can’t remember the exact date, please give a best estimate. | | | | | | | | *ENTER DATE* | | | | ____ / ____  mm / yy |

| C52 | What was the single most important thing that prompted you to get your last HIV test and receive the results? | Part of routine health care (e.g. antenatal, other health condition) | 1 |
| --- | --- | --- | --- |
|  |  | Referred for HIV testing by another health care provider | 2 |
|  |  | Conversation with or support from spouse/partner | 3 |
|  |  | Tested with or accompanied by spouse/partner | 4 |
|  |  | Spouse/partner recently tested positive | 5 |
|  |  | Conversation with or support from family member(s) or friend(s) | 6 |
|  |  | Tested with or accompanied by a family member(s) or friend(s) | 7 |
|  |  | Learned new information about HIV or HIV treatment that I didn’t know before | 8 |
|  |  | Learned about new changes in eligibility criteria for antiretroviral treatment (ART) (i.e. starting immediately upon testing positive, or at a higher CD4 count than before) | 9 |
|  |  | Began to experience symptoms of HIV/AIDS | 10 |
|  |  | Other (please specify) _____________ | 11 |
|  |  | Don’t know | 99 |

| C53 | How long did you wait between the time you first thought you should get an HIV test and the time you took the HIV test? | | 0-3 months | | | 1 |
| --- | --- | --- | --- | --- | --- | --- |
|  |  |  | 4-6 months | | | 2 |
|  |  |  | 7-12 months | | | 3 |
|  |  |  | 1-2 years | | | 4 |
|  |  |  | 2-5 years | | | 5 |
|  |  |  | More than 5 years | | | 6 |
|  |  |  | Decline to answer | | | 99 |
| C54 | I would like to ask you about the results of your most recent HIV test. A positive test result means that you have the virus that causes AIDS. I know that this is very sensitive information and I will not share your test results with anyone. If you are not prepared to discuss your results, you can refuse to answer this question.  What were the results of your **last** HIV test (or the last test from which you received results)? | | HIV Positive | | | 1 |
|  |  |  | HIV Negative [SKIP TO C60] | | | 0 |
|  |  |  | Don’t know [SKIP TO C60] | | | 8 |
|  |  |  | Decline to answer [SKIP TO C60] | | | 9 |
| C55 | | Are you currently seeing a health professional for your HIV infection (whether or not you started medications?) | Yes | | 1 | |
|  |  |  | No [SKIP TO C57] | | 2 | |
|  |  |  | Decline to answer [SKIP TO C57] | | 9 | |
| C56 | | How long did you wait between the time you tested positive and the time that you started seeing a health professional for your HIV infection (whether or not you started medications at the time)? | 0-3 months | | 1 | |
|  |  |  | 4-6 months | | 2 | |
|  |  |  | 7-12 months | | 3 | |
|  |  |  | 1-2 years | | 4 | |
|  |  |  | 2-5 years | | 5 | |
|  |  |  | More than 5 years | | 6 | |
|  |  |  | Decline to answer | | 99 | |
| C57 | Are you currently taking antiretroviral treatment (ART)? | | YES [SKIP TO C59] | | 1 | |
|  |  |  | NO | | 2 | |
|  |  |  | DK | | 3 | |
|  |  |  | Decline to answer | | 9 | |
| C58 | Do you have access* to antiretroviral treatment (ART), even if you are not currently taking it?  **In this context, access means that antiretroviral treatment is available and free or you can afford it.* | | YES | 1 | | |
|  |  |  | NO | 2 | | |
|  |  |  | DK | 3 | | |
|  |  |  | Decline to answer | 9 | | |
| C59 | Have you stopped taking your antiretroviral treatment (ART) at any point? | | YES | 1 | | |
|  |  |  | NO | 2 | | |

| C60 | [MEN] Are you circumcised? | YES [Proceed to C61 then skip to C64] | 1 |
| --- | --- | --- | --- |
|  |  | NO [SKIP TO C62] | 2 |
| C61 | [MEN] How old were you when you were circumcised? | AGE (in years): |  |
| C62 | [MEN] Are you considering getting circumcised? | YES | 1 |
|  |  | NO | 2 |
|  |  | DK | 9 |
| C63 | [MEN] If you wanted to get circumcised where would you prefer to go? | GOVERNMENT HOSPITAL OR CLINIC | 1 |
|  |  | PRIVATE HOSPITAL OR CLINIC | 2 |
|  |  | NGO/NGO CLINIC | 3 |
|  |  | TRADITIONAL HEALER/PROVIDER | 4 |
|  |  | Other (specify) | 5 |

| C64 | In the past 12 months: | | | YES | NO | |
| --- | --- | --- | --- | --- | --- | --- |
|  | 1. Did a medical provider test or examine you to see if you had a sexually transmitted infection other than HIV? | | | 1 | 2 | |
|  | 1. Did you provide a sputum sample for a TB test? | | | 1 | 2  [SKIP TO D.] | |
|  | 1. Were you told you were infected with TB? | | | 1 | 2 | |
|  | 1. Did you talk to a health care worker about family planning? | | | 1 | 2 | |
| C65 | Are you or your partner currently using a family planning method other than condoms? | | YES | | | 1 |
|  |  |  | NO | | | 2 |
| C66 | In the past 3 months have you received: | | YES | | | NO |
|  | 1. Condoms free? | | 1 | | | 2 |
|  | 1. Education about how to use condoms? | | 1 | | | 2 |
|  | 1. Personal lubricant for sex free? | | 1 | | | 2 |
| C67 | In the past 12 months, from where did you get condoms? | | YES | | | NO |
|  | 1. Public areas, such as bathrooms or clubs? | | 1 | | | 2 |
|  | 1. An outreach worker/peer educator? | | 1 | | | 2 |
|  | 1. Community organisations? | | 1 | | | 2 |
|  | 1. A clinic/hospital? | | 1 | | | 2 |
|  | 1. Stores or shops, such as petrol stations and pharmacies/chemists? | | 1 | | | 2 |
|  | 1. Street vendors? | | 1 | | | 2 |
|  | 1. Rural Health Motivators (RHMs)? | | 1 | | | 2 |
|  | 1. Bars, clubs, restaurants, or hotels? | | 1 | | | 2 |
|  | 1. Did not get condoms in the last 12 months | | 1 | | | 2 |
| C68 | Do you have a condom with you? Can I see it? | Yes, condom seen | | | | 1 |
|  |  | Yes, condom not seen | | | | 2 |
|  |  | No, condom not seen | | | | 3 |
| C69 | Do you currently have any of the following symptoms: | YES | | | | NO |
|  | 1. Discharge | 1 | | | | 2 |
|  | 1. Burning during urination | 1 | | | | 2 |
|  | 1. Genital sores | 1 | | | | 2 |
| C70 | How do you rate your chances of getting infected with HIV? | No chance | | | | 1 |
|  |  | Low | | | | 2 |
|  |  | Moderate | | | | 3 |
|  |  | High | | | | 4 |
|  |  | Already infected | | | | 9 |
| **PART V – RISK ASSESSMENT/BEHAVIOR** | | | | | | |
| C71 | Have you ever had sexual intercourse? | Yes | | | | 1 |
|  |  | No [SKIP TO C114] | | | | 2 |
| C72 | How old were you the first time you had sex? | AGE (in years) | | | |  |
| C73 | In the past 12 months, how many different sexual partners have you had?  IF 0, GO TO C91 and SKIP C95 to C111. | Number: | | | |  |
| C74 | [MEN] Of all the people you had sex with in the past 12 months, how many were girls between 15 and 19 years old (including a wife/partner)? 15-19-year-old girls look about the same age as middle-school or secondary school students.  (*Interviewer to urge respondents to give an estimate*) | *ENTER TOTAL IN LAST 12 MONTHS* | | | | _______ |
|  |  | DON’T KNOW | | | | 99 |
| C75 | [MEN] Of all the people you had sex within the past 12 months, how many were young women between 20 and 24 years old (including a wife/partner)? 20-24-year-old women look about the same age as post-high school students.  (*Interviewer to urge respondents to give an estimate*) | *ENTER TOTAL IN LAST 12 MONTHS* | | | | _______ |
|  |  | DON’T KNOW | | | | 99 |

| READ: Now I’m going to ask you some questions about your recent sexual activity. Let me assure you again that your answers are completely confidential and will not be told to anyone. If we should come to any question that you do not want to answer, just let me know and we will skip it and go to the next question | | | | |
| --- | --- | --- | --- | --- |
| **QUESTIONS and FILTERS** | | 1. **MOST RECENT** | 1. **SECOND-TO-LAST SEXUAL PARTNER** | 1. **THIRD-TO-LAST SEXUAL PARTNER** |
| C76 | What is the relationship of (your most recent/second most recent/third most recent) partner to you? | SPOUSE…………………..1  LIVE-IN PARTNER…….2  EX-SPOUSE ………..……3  EX-LIVE-IN PARTNER…………………4  STEADY PARTNER (NOT LIVING WITH RESPONDENT)…….….5  EX-STEADY PARTNER……………....6  CASUAL PARTNER………………..7  SEX WORKER………….8  OTHER…………………..9  SPECIFY___________ | SPOUSE…………………..1  LIVE-IN PARTNER…….2  EX-SPOUSE ………..……3  EX-LIVE-IN PARTNER…………………4  STEADY PARTNER (NOT LIVING WITH RESPONDENT)…….….5  EX-STEADY PARTNER……………....6  CASUAL PARTNER………………..7  SEX WORKER………….8  OTHER…………………..9  SPECIFY___________ | SPOUSE…………………..1  LIVE-IN PARTNER…….2  EX-SPOUSE ………..……3  EX-LIVE-IN PARTNER…………………4  STEADY PARTNER (NOT LIVING WITH RESPONDENT)…….….5  EX-STEADY PARTNER……………....6  CASUAL PARTNER………………..7  SEX WORKER………….8  OTHER…………………..9  SPECIFY___________ |
| C77 | What is the gender of this sexual partner? | Female…………………..1  Male………………………0 | Female…………………..1  Male………………………0 | Female…………………..1  Male………………………0 |
| C78 | How old is this sexual partner? If you don’t know for sure, please give a best guess. | [ ] years | [ ] years | [ ] years |
| C79 | About how old was she/he the first time you had sex with her/him? | [ ] years | [ ] years | [ ] years |
| C80 | Would you like to have a/another child with this partner? | YES ……………………...1  NO…………………..……2 | YES ……………………...1  NO…………………..……2 | YES ……………………...1  NO…………………..……2 |
| C81 | In the past 30 days about how many times did you have sexual intercourse with THIS PARTNER? | **________** | **________** | **________** |
| C82 | Are you still having sex with this partner? | YES ……………………...1  NO…………………..……2 | YES ……………………...1  NO…………………..……2 | YES ……………………...1  NO…………………..……2 |
| C83 | The last time you had sexual intercourse (with this most recent/second most recent/third most recent person) was a condom used? | YES ……………………...1  NO…………………..……2 | YES ……………………...1  NO…………………..……2 | YES ……………………...1  NO…………………..……2 |
| C84 | In the past 12 months when you had sexual intercourse (with this most recent/second most recent/third most recent person) was a condom used always, sometimes, or never? | ALWAYS………….……..1  SOMETIMES…….…….2  NEVER……………………3 | ALWAYS…………..…….1  SOMETIMES…….…….2  NEVER……………………3 | ALWAYS……………..….1  SOMETIMES……….….2  NEVER……………………3 |
| C85 | The last time you had sex with this partner, had **you** used any amount of alcohol just before sex? | YES ……………………...1  NO…………………..……2 | YES ……………………...1  NO…………………..……2 | YES ……………………...1  NO…………………..……2 |
| C86 | The last time you had sex with this partner, had **your partner** used any amount of alcohol just before sex? | YES ……………………...1  NO…………………..……2 | YES ……………………...1  NO…………………..……2 | YES ……………………...1  NO…………………..……2 |
| C87 | Have you talked to this partner about **his/her** HIV status? | YES ……………………...1  NO…………………..……2 | YES ……………………...1  NO…………………..……2 | YES ……………………...1  NO…………………..……2 |
| C88 | Have you talked to this partner about **your own** HIV status? | YES ……………………...1  NO…………………..……2 | YES ……………………...1  NO…………………..……2 | YES ……………………...1  NO…………………..……2 |
| C89 | Did you ever give this partner gifts or money in exchange for sex? | YES ……………………...1  NO…………………..……2 | YES ……………………...1  NO…………………..……2 | YES ……………………...1  NO…………………..……2 |
| C90 | Did you ever get gifts or money from this partner in exchange for sex? | YES ……………………...1  NO…………………..……2 | YES ……………………...1  NO…………………..……2 | YES ……………………...1  NO…………………..……2 |
| C91 | Did you ever physically force this partner to have sex when he/she did not want to? | YES ……………………...1  NO…………………..……2 | YES ……………………...1  NO…………………..……2 | YES ……………………...1  NO…………………..……2 |
| C92 | Did you ever hit, push, slap, punch, or kick this partner? | YES ……………………...1  NO…………………..……2 | YES ……………………...1  NO…………………..……2 | YES ……………………...1  NO…………………..……2 |

| C93 | Have you ever received money in exchange for sex? | | | | YES | | | | 1 | |
| --- | --- | --- | --- | --- | --- | --- | --- | --- | --- | --- |
|  |  |  |  |  | NO [SKIP TO C97] | | | | 2 | |
| C94 | When was the last time you received money in exchange for sex? | | | | Last 3 months | | | | 1 | |
|  |  |  |  |  | Last 6 months | | | | 2 | |
|  |  |  |  |  | Last 12 months | | | | 3 | |
|  |  |  |  |  | Longer than 12 months | | | | 4 | |
| C95 | Did you use a condom the last time you got paid for sex? | | | | YES | | | | 1 | |
|  |  |  |  |  | NO | | | | 2 | |
| C96 | How old were you the first time you were paid for sex? | | | | AGE (in years): | | | |  | |
| C97 | Have you ever received gifts (including goods or favors) in exchange for sex in the last 12 months? | | | | YES | | | | 1 | |
|  |  |  |  |  | NO [SKIP TO C99] | | | | 2 | |
| C98 | If yes, in exchange for: | | | | YES | | | | NO | |
|  | 1. Airtime? | | | | 1 | | | | 2 | |
|  | 1. Food? | | | | 1 | | | | 2 | |
|  | 1. Clothes/shoes? | | | | 1 | | | | 2 | |
|  | 1. Grades/good marks in school? | | | | 1 | | | | 2 | |
|  | 1. Rent? | | | | 1 | | | | 2 | |
|  | 1. Other ___________? | | | | 1 | | | | 2 | |
| C99 | Have you ever paid money in exchange for sex in the past 12 months? | | | | YES | | | | 1 | |
|  |  |  |  |  | NO | | | | 2 | |
| C100 | Have you ever given gifts (including goods or favors) in exchange for sex in the last 12 months? | | | | YES | | | | 1 | |
|  |  |  |  |  | NO [SKIP TO C100] | | | | 2 | |
| C101 | If yes, in exchange for: | | | | YES | | | | NO | |
|  | 1. Airtime? | | | | 1 | | | | 2 | |
|  | 1. Food? | | | | 1 | | | | 2 | |
|  | 1. Clothes/shoes? | | | | 1 | | | | 2 | |
|  | 1. Grades/good marks in school? | | | | 1 | | | | 2 | |
|  | 1. Rent? | | | | 1 | | | | 2 | |
|  | 1. Other ___________? | | | | 1 | | | | 2 | |
| C102 | How many sexual partners have you had: | | | | TOTAL in the last 4 weeks | | | |  | |
|  |  |  |  |  | NEW in the last 4 weeks | | | |  | |
|  |  |  |  |  | TOTAL in the last 6 months | | | |  | |
|  |  |  |  |  | NEW in the last 6 months | | | |  | |
|  |  |  |  |  | TOTAL in the last 12 months | | | |  | |
| C103 | Have you had any NEW sexual partners in the last 12 months? | | | | YES | | | | 1 | |
|  |  |  |  |  | NO [SKIP TO C102] | | | | 2 | |
| C104 | In the past 12 months, at what kinds of places have you met new sex partners: | | | | YES | | | | NO | |
|  | 1. The street? | | | | 1 | | | | 2 | |
|  | 1. A bar or club? | | | | 1 | | | | 2 | |
|  | 1. Taxi/bus rank? | | | | 1 | | | | 2 | |
|  | 1. A school? | | | | 1 | | | | 2 | |
|  | 1. Church? | | | | 1 | | | | 2 | |
|  | 1. Friend’s house? | | | | 1 | | | | 2 | |
|  | 1. My workplace? | | | | 1 | | | | 2 | |
|  | 1. Shopping Mall? | | | | 1 | | | | 2 | |
|  | 1. Other? Specify _____________________ | | | | 1 | | | | 2 | |
| C105 | In the past 12 months, have you met new sex partners in: | | | | YES | | | | NO | |
|  | 1. This inkhundla [NAME IT] | | | | 1 | | | | 2 | |
|  | 1. This region [NAME IT] | | | | 1 | | | | 2 | |
|  | 1. Elsewhere in Swaziland | | | | 1 | | | | 2 | |
|  | 1. Another country: _____________________________ | | | | 1 | | | | 2 | |
| C106 | Do you currently live with your sex partner? | | | | YES | | | | 1 | |
|  |  |  |  |  | NO | | | | 2 | |
| C107 | Do you believe that your main partner has had sex with another partner in the past 12 months? | | | | YES | | | | 1 | |
|  |  |  |  |  | NO | | | | 2 | |
| C108 | In the last 12 months, have you had sex with: | | | | Men? | | | | 1 | |
|  |  |  |  |  | Women? | | | | 2 | |
| C109 | What is the age difference of your last male partner? | | | | 10 or more years older | | | | 1 | |
|  |  |  |  |  | 5 to 9 years older | | | | 2 | |
|  |  |  |  |  | 0 to 4 years older | | | | 3 | |
|  |  |  |  |  | 0 to 4 years younger | | | | 4 | |
|  |  |  |  |  | 5 to 9 years younger | | | | 5 | |
|  |  |  |  |  | 10 or more years younger | | | | 6 | |
|  |  |  |  |  | Never had a male partner | | | | 7 | |
| C110 | What is the age difference of your last female partner? | | | | 10 or more years older | | | | 1 | |
|  |  |  |  |  | 5 to 9 years older | | | | 2 | |
|  |  |  |  |  | 0 to 4 years older | | | | 3 | |
|  |  |  |  |  | 0 to 4 years younger | | | | 4 | |
|  |  |  |  |  | 5 to 9 years younger | | | | 5 | |
|  |  |  |  |  | 10 or more years younger | | | | 6 | |
|  |  |  |  |  | Never had a female partner | | | | 7 | |
| C111 | What is the age difference of your main or live-in partner? | | | | 10 or more years older | | | | 1 | |
|  |  |  |  |  | 5 to 9 years older | | | | 2 | |
|  |  |  |  |  | 0 to 4 years older | | | | 3 | |
|  |  |  |  |  | 0 to 4 years younger | | | | 4 | |
|  |  |  |  |  | 5 to 9 years younger | | | | 5 | |
|  |  |  |  |  | 10 or more years younger | | | | 6 | |
|  |  |  |  |  | Do not have a main partner | | | | 7 | |
| C112 | In general, what is the age of all male partners in the past year? | | | | Mostly younger | | | | 1 | |
|  |  |  |  |  | Mostly older | | | | 2 | |
|  |  |  |  |  | Both younger and older | | | | 3 | |
|  |  |  |  |  | Mostly about the same age | | | | 4 | |
|  |  |  |  |  | No male partners in the past year. | | | | 5 | |
| C113 | In general, what is the age of all female partners in the past year? | | | | Mostly younger | | | | 1 | |
|  |  |  |  |  | Mostly older | | | | 2 | |
|  |  |  |  |  | Both younger and older | | | | 3 | |
|  |  |  |  |  | Mostly about the same age | | | | 4 | |
|  |  |  |  |  | No female partners in the past year. | | | | 5 | |
| C114 | How often do you drink alcohol? | | | | Never [SKIP TO C115] | | | | 1 | |
|  |  |  |  |  | Monthly or less | | | | 2 | |
|  |  |  |  |  | 2-4 times per month | | | | 3 | |
|  |  |  |  |  | 2-3 times per week | | | | 4 | |
|  |  |  |  |  | 4 or more times per week | | | | 5 | |
| C115 | Typically, when you drink alcohol, how many drinks do you have? | | | | 1-2 | | | | 1 | |
|  |  |  |  |  | 3-4 | | | | 2 | |
|  |  |  |  |  | 5-6 | | | | 3 | |
|  |  |  |  |  | 7-9 | | | | 4 | |
|  |  |  |  |  | 10+ | | | | 5 | |
| C116 | How often do you drink 6 or more alcoholic drinks on one occasion? | | | | Never | | | | 1 | |
|  |  |  |  |  | Less than monthly | | | | 2 | |
|  |  |  |  |  | Monthly | | | | 3 | |
|  |  |  |  |  | Weekly | | | | 4 | |
|  |  |  |  |  | Daily or almost daily | | | | 5 | |
| C117 | In the past 6 months have you used: | | | | YES | | | | NO | |
|  | 1. Marijuana | | | | 1 | | | | 2 | |
|  | 1. Heroin | | | | 1 | | | | 2 | |
|  | 1. Cocaine | | | | 1 | | | | 2 | |
|  | 1. Crack | | | | 1 | | | | 2 | |
|  | 1. Methamphetamines | | | | 1 | | | | 2 | |
|  | 1. Other opioids | | | | 1 | | | | 2 | |
|  | 1. Solvents/glue | | | | 1 | | | | 2 | |
|  | 1. Hallucinogens | | | | 1 | | | | 2 | |
| **PART VI –VULNERABILITY AND VIOLENCE** | | | | | | | | | | |
| C118 | Have you been jailed or in prison in the last 12 months? | | | | YES | | | | 1 | |
|  |  |  |  |  | NO | | | | 2 | |
| C119 | Have you slept outside or been homeless in the last 12 months? | | | | YES | | | | 1 | |
|  |  |  |  |  | NO | | | | 2 | |
| C120 | In the last 30 days have you or your family gone a whole day without eating anything because there was not enough food? | | | | YES | | | | 1 | |
|  |  |  |  |  | NO | | | | 2 | |
| C121 | Have you been physically hurt (hit, pushed, slapped, kicked, punched) by a sex partner in the last 12 months? | | | | YES | | | | 1 | |
|  |  |  |  |  | NO | | | | 2 | |
| C122 | Have you ever been forced to have sex (physically forced, coerced to have sex, or penetrated with an object) when you did not want? | | | | YES | | | | 1 | |
|  |  |  |  |  | NO | | | | 2 | |
| **PART VI – GENDER AND RELATIONSHIP DYNAMICS** | | | | | | | | | | |
| READ: For the next few questions, we are asking if you agree, partially agree, or do not agree with a statement. | | | | | | | | | | |
|  |  | | | | AGREE | | PARTIALLY AGREE | | DO NOT AGREE | |
| C123 | A woman should tolerate violence to keep her family together. | | | | 1 | | 2 | | 3 | |
| C124 | It is the man who decides what type of sex to have. | | | | 1 | | 2 | | 3 | |
| C125 | Men are always ready to have sex. | | | | 1 | | 2 | | 3 | |
| C126 | Women who carry condoms on them are easy. | | | | 1 | | 2 | | 3 | |
| C127 | It is a woman’s responsibility to avoid getting pregnant. | | | | 1 | | 2 | | 3 | |
| C128 | Only when a woman has a child is she a real woman. | | | | 1 | | 2 | | 3 | |
| C129 | A real man produces a male child. | | | | 1 | | 2 | | 3 | |
| C130 | Changing diapers, giving a bath, and feeding kids are the mother’s responsibility. | | | | 1 | | 2 | | 3 | |
| C131 | A woman’s role is taking care of her home and family. | | | | 1 | | 2 | | 3 | |
| C132 | The husband should decide to buy the major household items. | | | | 1 | | 2 | | 3 | |
| C133 | A man should have the final word about decisions in his home. | | | | 1 | | 2 | | 3 | |
| C134 | A woman should obey her husband in all things. | | | | 1 | | 2 | | 3 | |
| C135 | A woman who is unfaithful needs to be put in her place. | | | | 1 | | 2 | | 3 | |
| C136 | A man is expected to discipline his woman. | | | | 1 | | 2 | | 3 | |
| C137 | A man needs more than one woman. | | | | 1 | | 2 | | 3 | |
| C138 | For men, getting sick is a sign of weakness. | | | | 1 | | 2 | | 3 | |
| C139 | Health clinics are for women and children. | | | | 1 | | 2 | | 3 | |
| C140 | A man shouldn’t go to the doctor unless his situation is serious. | | | | 1 | | 2 | | 3 | |
| **PART VII - EXPOSURE TO DREAMS PROGRAMMING** | | | | | | | | | | |
| C141 | | Have you heard of the DREAMS Initiative? | | | | YES 1 | | | | |
|  |  |  |  |  |  | NO 2 | | | | |
|  |  |  |  |  |  | Don’t know 99 | | | | |
| C142 | | Have you seen this logo in the last year? [Show picture of DREAMS logo, in color] | | | | YES 1 | | | | |
|  |  |  |  |  |  | NO 2 | | | | |
|  |  |  |  |  |  | Don’t know 99 | | | | |
| *We would like to learn about your experiences in the past year with community or group meetings about HIV that have taken place nearby. For each question, please consider only those experiences you have had in the past year – or since [MONTH].* | | | | | | | | | | |
| C143 | | Since [MONTH], have you attended any community or group meetings about HIV around here? This would be a meeting with at least 3 other people. | | | | Yes 1  No 2 [SKIP T0 C149] | | | | |
| C144 | | Since [MONTH], how many community meetings or group meetings about HIV have you gone to? (If you don’t know the exact number, please give a best guess) | | | | NUMBER of meetings [_______] | | | | |
| C145 | | Please tell me whether any of the following groups or organizations arranged these community meetings or dialogues about HIV that you attended? |  | | | YES | | NO | | DK |
|  |  |  | 1. DREAMS Initiative | | | 1 | | 2 | | 8 |
|  |  |  | 1. Bantwana | | | 1 | | 2 | | 8 |
|  |  |  | 1. Compassionate Swaziland | | | 1 | | 2 | | 8 |
|  |  |  | 1. Khulisa | | | 1 | | 2 | | 8 |
|  |  |  | 1. World Vision | | | 1 | | 2 | | 8 |
|  |  |  | 1. Young Heroes | | | 1 | | 2 | | 8 |
|  |  |  | 1. PEPFAR / USAID | | | 1 | | 2 | | 8 |
|  |  |  | 1. NERCHA | | | 1 | | 2 | | 8 |
|  |  |  | 1. Ministry of Health | | | 1 | | 2 | | 8 |
|  |  |  | 1. Other (please name it):   __________________ | | | 1 | | 2 | | 8 |
| C146 | | Did you discuss any of the following topics in these community or group meetings, since [MONTH]? |  | | | YES | | NO | | DK |
|  |  |  | 1. Preventing HIV transmission | | | 1 | | 2 | | 8 |
|  |  |  | 1. Gender norms / gender-based violence / healthy relationships | | | 1 | | 2 | | 8 |
|  |  |  | 1. HIV counseling and testing | | | 1 | | 2 | | 8 |
|  |  |  | 1. HIV care and treatment | | | 1 | | 2 | | 8 |
|  |  |  | 1. Voluntary medical male circumcision (VMMC) | | | 1 | | 2 | | 8 |
|  |  |  | 1. Other (please name it):   __________________ | | | 1 | | 2 | | 8 |
| C147 | | While participating in these community or group meetings, since [MONTH], did you receive a referral to an HIV-related service such as HIV counseling and testing (HCT), HIV care or treatment, or voluntary medical male circumcision (VMMC)? |  | | | YES and used referral | | YES but didn’t use referral | | NO |
|  |  |  | 1. HIV counseling and testing (HCT) | | | 1 | | 2 | | 8 |
|  |  |  | 1. HIV care and treatment | | | 1 | | 2 | | 8 |
|  |  |  | 1. Voluntary medical male circumcision (VMMC) | | | 1 | | 2 | | 8 |
|  |  |  | 1. Other (please name it):   __________________ | | | 1 | | 2 | | 8 |
| C148 | | The last time you attended a community or group meeting about HIV, about how many other people also attended that meeting?  (If you don’t know the exact number, please give a best guess) | | | | NUMBER OF OTHER ATTENDEES  [_______]  Don’t remember 99 | | | | |
| *Next, we would like to learn about your experiences in the past year with one-on one discussions about HIV with someone from a program, where it was just you and that person, and maybe one or two other people, discussing together. For each question, please consider only those experiences you have had in the past year – or since [MONTH].* | | | | | | | | | | |
| C149 | | Since [MONTH] have you met one-on one, or in a group with just one or two other people, with someone who is working for a program about HIV? | | | | Yes 1  No 2 [SKIP TO C154] | | | | |
| C150 | | Since [MONTH] how many times did you meet with someone who was working for a program about HIV? (If you can’t remember exactly, please give a best guess) | | | | NUMBER OF TIMES  [__________] | | | | |
| C151 | | Please tell me what groups or organizations the person / people you met with one-on-one worked for? |  | | | YES | | NO | | DK |
|  |  |  | 1. DREAMS Initiative | | | 1 | | 2 | | 8 |
|  |  |  | 1. Bantwana | | | 1 | | 2 | | 8 |
|  |  |  | 1. Compassionate Swaziland | | | 1 | | 2 | | 8 |
|  |  |  | 1. Khulisa | | | 1 | | 2 | | 8 |
|  |  |  | 1. World Vision | | | 1 | | 2 | | 8 |
|  |  |  | 1. Young Heroes | | | 1 | | 2 | | 8 |
|  |  |  | 1. PEPFAR/USAID | | | 1 | | 2 | | 8 |
|  |  |  | 1. NERCHA | | | 1 | | 2 | | 8 |
|  |  |  | 1. Ministry of Health | | | 1 | | 2 | | 8 |
|  |  |  | 1. Other (please name it):   __________________ | | | 1 | | 2 | | 8 |
| C152 | | Did you discuss any of the following topics in these one-on-one meetings, since [MONTH]? |  | | | YES | | NO | | DK |
|  |  |  | 1. Preventing HIV transmission | | | 1 | | 2 | | 8 |
|  |  |  | 1. Gender norms / gender-based violence / healthy relationships | | | 1 | | 2 | | 8 |
|  |  |  | 1. HIV counseling and testing (HCT) | | | 1 | | 2 | | 8 |
|  |  |  | 1. HIV care and treatment | | | 1 | | 2 | | 8 |
|  |  |  | 1. Voluntary medical male circumcision (VMMC) | | | 1 | | 2 | | 8 |
|  |  |  | 1. Other (please name it):   __________________ | | | 1 | | 2 | | 8 |
| C153 | | While participating in these one-on-one meetings, since [MONTH], did you receive a referral to the following HIV-related services? |  | | | YES and have used the referral | | YES but haven’t used the referral | | NO |
|  |  |  | 1. HIV counseling and testing (HCT) | | | 1 | | 2 | | 8 |
|  |  |  | 1. HIV care and treatment | | | 1 | | 2 | | 8 |
|  |  |  | 1. Voluntary medical male circumcision (VMMC) | | | 1 | | 2 | | 8 |
|  |  |  | 1. Other (please name it):   __________________ | | | 1 | | 2 | | 8 |
| *Next, we would like to learn about HIV-related services you received, and what organization provided each of these services. For each question, please consider only those services you have used in the past year – or since [MONTH].* | | | | | | | | | | |
| *A. Have you received this service since [MONTH]?*  *Yes 1*  *No 2 [SKIP TO NEXT QUESTION]*  *Don’t know 8 [SKIP TO NEXT QUESTION]* | | | | *B.* Please tell me what group or organization provided this service? (Select all that apply)  *DREAMS Initiative 1*  Bantwana 2  Compassionate Swaziland 3  Khulisa 4  World Vision 5  Young Heroes 6  *PEPFAR / USAID 7*  *NERCHA 8*  *Ministry of Health 9*  *Other __________________ 10*  *Don’t know/don’t remember 99* | | | | | | |
| C154 HIV counseling and testing (HCT) at a clinic/facility | | | |  | | | | | | |
| C155 HIV counseling and testing (HCT) at home | | | |  | | | | | | |
| C156 HIV counseling and testing (HCT) at a community venue (such as a bar, nightclub, or taxi rank) | | | |  | | | | | | |
| C157 HIV counseling and testing (HCT) at your workplace | | | |  | | | | | | |
| C158 HIV care and/or treatment services | | | |  | | | | | | |
| C159 Voluntary medical male circumcision (VMMC) | | | |  | | | | | | |
| C160 Other service (please name it): ___________________ | | | |  | | | | | | |
| **THANK YOU!** | | | | | | | | | | |
